# Prognostic significance and immune microenvironment infiltration patterns of hypoxia and endoplasmic reticulum stress-related genes in gastric cancer

**DOI:** 10.3389/fonc.2025.1542740

**Published:** 2025-02-21

**Authors:** Libin Li, Yizhi Liang, Wenji Xu

**Affiliations:** Department of Gastroenterology, The Second Affiliated Hospital of Fujian Medical University, Quanzhou, Fujian, China

**Keywords:** gastric cancer, hypoxia, endoplasmic reticulum stress, biomarkers, prognostic model, immune infiltration

## Abstract

**Background:**

Gastric cancer (GC) is a prevalent malignant neoplasm within the digestive system, accounting for approximately 740,000 deaths globally each year, significantly impacting patients' quality of life and survival rates. The objective of this investigation was to elucidate the expression patterns of Hypoxia and Endoplasmic Reticulum Stress-related Differentially Expressed Genes (HERSRDEGs) in GC and their association with prognostic outcomes of the patients.

**Methods:**

Utilizing The Cancer Genome Atlas (TCGA) and Gene Expression Omnibus (GEO) databases, GC datasets were retrieved, and standard normalization was performed. Differential expression analysis was conducted using DESeq2, while somatic mutations and copy number variations were examined using maftools and GISTIC2.0. Spearman's correlation assessed the interplay between HERSRDEGs across datasets. Functional enrichment analyses were carried out using clusterProfiler for Gene Ontology (GO) and Kyoto Encyclopedia of Genes and Genomes (KEGG) pathways, alongside Gene Set Enrichment Analysis (GSEA). A prognostic risk model was obtained by utilizing univariate Cox regression analysis with a survival R package. We employed RT-qPCR to validate the mRNA expression levels of five model genes that impact the prognostic risk of gastric cancer in human gastric adenocarcinoma tissues.

**Results:**

The acquired data revealed 19 HERSRDEGs including *ANGPT2, CXCL8*, and *AURKA* exhibiting significant variation in expression between GC and controls. In the Cox regression analysis, a total of five genes—*ANGPT2, CD36, EGR1, NOX4, TLR2*—emerged as statistically significant, correlating strongly with overall survival. A LASSO regression model featuring *ANGPT2, CD36*, and *NOX4* yielded a risk score formula capable of predicting patient outcomes. Furthermore, multivariate Cox regression analysis highlighted RiskScore, age and stage as significant survival predictors. The analysis of immune infiltration revealed notable differences in the populations of immune cells, such as Natural Killer cells and T-helper cells, when comparing high-risk and low-risk groups.

**Conclusion:**

In conclusion, this investigation elucidates the involvement of HERSRDEGs in GC progression and their potential as prognostic biomarkers.

## 1 Introduction

Gastric cancer (GC) ranks as the fifth most common cancer type and the fourth leading cause of cancer-related mortality worldwide, accounting for around 768,793 deaths and 1,089,103 new diagnoses in the year 2020 (1). Despite progress in adjuvant therapies and surgical techniques over the years, the prognostic outcomes for GC patients remain dismal, particularly in advanced stages, with a five-year survival rate of under 30% and median survival of less than 1 year (2). This high mortality rate is largely attributed to late diagnosis, rapid disease progression, and resistance to current treatment modalities (3). These challenges emphasize the necessity for novel biomarkers and therapeutic targets aimed at enhancing early detection, prognosis, and treatment outcomes in GC.

Hypoxia and endoplasmic reticulum stress (ERS) are phenotypic hallmarks associated with the tumor microenvironment and have been linked to the pathogenesis and progression of diverse cancers, including GC (4, 5). Protein misfolding within the endoplasmic reticulum results in the buildup of improperly folded proteins and activates the unfolded protein response (UPR). This response has developed as a mechanism to maintain an effective protein-folding environment within the endoplasmic reticulum. The activation of both ERS and UPR has been observed across diverse human cancers. Hypoxia-inducible factors (HIFs) and the UPR pathways are pivotal in the cellular adaptation to hypoxia and ERS, respectively, and their dysregulation has been linked to oncogenesis, metastasis, and treatment resistance (6, 7). Notably, hypoxia and Endoplasmic Reticulum Stress-Related Differentially Expressed Genes (HERSRDEGs) have shown promise as prognostic indicators and therapeutic targets in specific malignancies, including breast and colorectal cancer (8, 9), suggesting their significant research potential in GC.

Our study sought to investigate the involvement of HERSRDEGs in the pathogenesis and development of GC, as well as to establish a prognostic risk model. To accomplish this goal, GC datasets were retrieved from TCGA and GEO databases and processed, differential gene expression analysis was performed, and somatic mutations and copy number variations were assessed. Correlation analyses, gene-set enrichment analysis (GSEA), functional enrichment, and immune infiltration assessments were conducted to elucidate the clinical significance of HERSRDEGs in GC. Our research offers considerable insights into the influence of hypoxia and ERS in gastric carcinogenesis and proposes a novel prognostic model based on HERSRDEGs, potentially guiding future clinical interventions for GC individuals.

## 2 Materials and methods

### 2.1 Downloading of data

The dataset pertaining to Stomach Adenocarcinoma from The Cancer Genome Atlas (TCGA-STAD) was obtained from the TCGA database using the R package TCGAbiolinks (10) and utilized as the test set. After excluding samples lacking prognostic data, sequencing data in Counts format were acquired from 371 GC samples that possessed prognostic information, along with 32 control samples. Following this, the data underwent normalization to the Fragments Per Kilobase per Million (FPKM) standard, while pertinent clinical information was sourced from the UCSC Xena database (11). Detailed information can be found in **Supplementary Table S7**. It is important to note that the TCGA-STAD database does not include specific information about chemotherapy or other treatments. Hence, this information is not shown in **Supplementary Table S7**. The GC datasets GSE142000, and GSE118897 (12) were downloaded using the R program GEOquery (13) accessed at the GEO database (14) (https://www.ncbi.nlm.nih.gov/geo/) as a validation set for further assessment. The samples analyzed in this investigation, obtained from GSE142000 and GSE118897, were exclusively obtained from Homo sapiens and derived from gastric tissue. The gene expression data were generated using the chip platforms of GPL23227 and GPL16686 for GSE142000 and GSE118897, respectively, as detailed in **Supplementary Table S8**. GSE142000 comprised 7 GC samples and 6 control samples, and GSE118897 comprised 10 GC samples and 10 control samples. The samples from both datasets were utilized in this investigation for comprehensive assessment.

We collected candidate genes from multiple databases and screened and validated these genes through a series of bioinformatics analyses, aiming to identify HERSRDEGs associated with gastric cancer.

First, we searched the GeneCards database (15) (https://www.genecards.org/) and MSigDB database (16) (https://www.gsea-msigdb.org/gsea/msigdb) with the keywords “Hypoxia” and “Endoplasmic Reticulum Stress”, and selected the gene “Protein Coding”. Retained “Protein Coding” genes with a “Relevance Score > 4” and “Protein Coding” genes with a “Relevance Score > 2”, resulting in 239 hypoxia-related genes (HRGs) and 603 Endoplasmic Reticulum Stress-Related Genes (ERSRGs). Subsequently, 397 HRGs and 26 ERSRGs were extracted by searching related literature in PubMed database, respectively (17, 18). After combining and removing duplicate genes, we finally identified 624 HRGs (as detailed in **Supplementary Table S1**) and 608 ERSRGs (as detailed in **Supplementary Table S2**), and took their intersection to obtain 103 hypoxia and endoplasmic reticulum stress-related genes (HERSRGs) with detailed information presented in **Supplementary Table S3**.

The datasets GSE142000 and GSE118897 were standardized using the R tool limma (19). This method included standardizing and normalizing the annotation probes. Subsequently, the datasets GSE142000, GSE118897, and the GC dataset (TCGA-STAD) were utilized to identify the intersection genes. Only the expression matrix of these intersected genes was retained for further analysis.

### 2.2 Assessing gastric cancer-related hypoxia & endoplasmic reticulum stress-related differentially expressed genes

In the screening of differentially expressed genes(DEGs), we used the R package DESeq2 (20) to analyze GC samples and control samples in the TCGA-STAD dataset. We set |logFC| > 1 and adj.P< 0.05 as the screening criteria to ensure that the selected genes had significant differences. In integrating the data, we ensured that only the overlapping genes present in all datasets were included to improve the reliability of subsequent analyses. Using the R package ggplot2, volcano plots were created to illustrate the variance analysis results.

In order to filter HERSRDEGs associated with GC, the DEGs from the GC dataset (TCGA-STAD) with |logFC| > 1 and adj. P< 0.05 were intersected with 103 HERSRGs. The intersection set of HERSRDEGs was visualized by plotting a Venn diagram Additionally, a heat map was generated utilizing the R package pheatmap, while chromosomal localization maps were constructed using RCircos (21) was employed to create chromosomal localization maps.

### 2.3 Somatic mutation, copy number variation analysis

Using “Masked Somatic Mutation” data as the Somatic Mutation (SM) data, samples from the GC dataset (TCGA-STAD) were examined for SM. These data were derived from TCGA, and preprocessed by VarScan software. Finally, R maftools (22) was utilized to visualize the SM landscape, providing insights into the mutational profile of GC samples.

The “Masked Copy Number Segment” dataset was selected as the source of Copy Number Variation (CNV) data for the analysis of CNVs in GC samples extracted from the GC dataset (TCGA-STAD). The data were acquired from TCGA. The GISTIC2.0 (23) analysis was performed on the downloaded and processed CNV segments, and all the default parameters were utilized.

### 2.4 Correlation analysis

To investigate the relationship among HERSRDEGs more comprehensively, the Spearman algorithm was employed to assess HERSRDEGs within the GC dataset (TCGA-STAD). Additionally, correlation analysis was performed to evaluate the association between the expression levels of the GSE142000 and GSE118897 datasets. The findings of the analysis were illustrated using the R package pheatmap, which was utilized to generate the correlation heatmap. Subsequently, the positive and negative correlation of top1 related HERSRDEGs were screened. The correlation scatter plot was generated using the R package ggplot2. Correlation coefficients were interpreted as follows: an absolute value lower than 0.3 indicated zero or weak correlation, 0.3-0.5 signified weak correlation, 0.5-0.8 signified moderate correlation, and above 0.8 signified strong correlation.

### 2.5 Gene ontology and pathway (KEGG) enrichment analysis

Gene Ontology (GO) analysis (24) is a widely employed measure for studying functional enrichment on a large scale, encompassing Biological Process (BP), Cell Component (CC), and Molecular function (MF). The Kyoto Encyclopedia of Genes and Genomes (KEGG) (25) is a commonly utilized resource that provides an extensive array of data pertaining to diseases, genomes, biological pathways and drugs. GO and KEGG enrichment analyses of HERSRDEGs were conducted through R clusterProfiler (26). A P-value of less than 0.05, along with a false discovery rate (FDR) value, also referred to as the Q value, lower than 0.25, was considered indicative of statistical significance. The Benjamini-Hochberg (BH) method was employed for the adjustment of P-values.

### 2.6 Gene Set Enrichment Analysis

GSEA (27) is employed to examine how genes in a predefined gene set are distributed within a gene table sorted by their correlation with a phenotype, thereby identifying their impact on the phenotype. In this investigation, the genes in the GC dataset (TCGA-STAD) were first ordered per the logFC values, and GSEA was then conducted on these genes using R clusterProfiler. The parameters utilized in GSEA included a seed value of 2020, 1000 computations, a minimum of 10, and a maximum of 500 genes in each gene set. The MSigDB Database (16) was accessed to acquire the gene set c2.cp.all.v2022.1.Hs.symbols.gmt [All Canonical Pathways] (3050) which was employed for GSEA. The screening criterion for GSEA was set as P value < 0.05.

### 2.7 Establishment of a prognostic risk model for gastric cancer

To establish the prognostic risk model in the GC dataset (TCGA-STAD), R survival (28) was employed to conduct univariate Cox regression analysis utilizing clinical information to evaluate the effect of HERSRDEGs on prognosis. We selected prognostically relevant differentially expressed genes (HERSRDEGs) in a univariate Cox regression analysis and identified those genes with a P-value of less than 0.10 by calculating the P-value of each gene relative to overall survival (OS). The selection of these genes provides a basis for subsequent modeling. In order to further screen genes with a significant influence on prognostic risk factors, we used Least Absolute Shrinkage and Selection Operator (LASSO) regression analysis and set the cycle number to 10 to obtain the model genes for the prognostic risk model. LASSO analysis was executed via R glmnet (29).Finally, the RiskScore was determined using the risk coefficient derived from LASSO regression analysis, as represented in the following formula:

Equation 1:


$$\text{risk}Score\;=\;\sum_{i}Coefficient\;\left(gene_{i}\right)*mRNA\;Expression\;\left(gene_{i}\right)$$

According to **Equation 1**, this equation facilitates the computation of the risk score by aggregating the products of the gene coefficients and their respective mRNA expression levels.

### 2.8 Prognostic analysis of gastric cancer prognostic risk model

The Time-dependent Receiver Operating Characteristic (ROC) Curve (30) serves as a schematic analysis instrument frequently employed for model selection, threshold optimization, and performance assessment. The R package survivalROC was used to draw time-dependent ROC curves and compute the Area Under the Curve (AUC) based on RiskScore and overall survival (OS). The survival outcomes for 1, 3, and 5 years of GC individuals from the dataset (TCGA-STAD) were predicted by assessing the performance of our model using the AUC of the ROC curve. This value was generally found to be between 0.5 and 1. An increased value (approaching 1) signifies enhanced diagnostic efficacy. An AUC value greater than 0.5 indicates a correlation between the gene expression and the event’s occurrence. To analyze the variation in OS across the high- and low-risk groups of the GC dataset (TCGA-STAD), the R package survival was utilized for Kaplan-Meier (KM) curve (31) analysis. KM curves were drawn based on the RiskScore.

The outcomes of the univariate and multivariate Cox regression analyses, which encompassed both the expression of RiskScore and relevant clinical data, were illustrated through a Forest Plot. To illustrate the relationship between RiskScore and clinical information included in the multivariate Cox regression model, a nomogram was established. A Nomogram (32) is a graphical depiction of the functional relationship between multiple independent variables. It achieves this by presenting a group of non-overlapping line segments in a rectangular coordinate framework. The R package ggDCA was employed to establish a Nomogram based on the outcomes of the multivariate Cox regression analysis.

The Calibration Curve serves as a crucial instrument for evaluating the predictive precision of a model, allowing for a comparison between the true outcome probabilities and the predictions generated by the model across diverse scenarios. It helps evaluate the model’s calibration, or how closely the predicted probabilities match the observed probabilities. The precision and distinguishing capacity of the prognostic risk model based on RiskScore was examined by generating the Calibration Curve via Calibration analysis. Decision Curve Analysis (DCA) (33) is a method employed to assess clinical prediction models, molecular biomarkers, and diagnostic tests. R ggDCA package was utilized to generate a DCA plot based on RiskScore to examine the discriminative capacity and accuracy of the prognostic risk model for GC.

### 2.9 Validation of differential expression

Initially, the GC samples from the TCGA-STAD dataset were categorized into high-risk and low-risk cohorts based on the median expression level of RiskScore obtained from the GC prognostic risk model. Subsequently, a comparative analysis of the model gene expression across these two risk groups was conducted in order to assess the variation between them. This assessment involved plotting a group comparison map visualizing the expression levels of these model genes. Finally, R pROC was utilized to establish the ROC Curve of model genes and compute the corresponding AUC. This analysis aimed to examine the diagnostic capacity of model gene expression in terms of predicting the occurrence of GC. Generally, the area beneath the ROC curve demonstrates values between 0.5 and 1, where elevated values indicate superior diagnostic efficacy. AUC values within the range of 0.5 and 0.7 signified diminished accuracy, whereas moderate accuracy was associated with AUC values within the range of 0.7 and 0.9, with values above 0.9 signifying high accuracy.

### 2.10 Gene set variation analysis

Gene Set Variation Analysis (GSVA) (34), is an unsupervised non-parametric analysis approach utilized for evaluating gene set enrichment data derived from microarray nuclear transcriptome data. This approach enables the assessment of whether distinct pathways exhibit enrichment across various samples. The process involves accessing the gene set data from MSigDB and the GC samples from the TCGA–STAD dataset. By utilizing the MSigDB and TCGA–STAD dataset, GSVA enables the exploration of the functional enrichment variation across high- and low-risk cohorts. The screening criteria of GSVA was set as adj.P < 0.05 and the BH approach was employed for P value adjustment.

### 2.11 Immune infiltration analysis

Single-sample gene-set enrichment analysis (ssGSEA) (35) is a technique employed to measure the relative levels of immune cell infiltration in specific samples. The enrichment scores derived from ssGSEA were employed to assess the relative presence of immune cell infiltrates within each individual sample, thereby generating an immune cell infiltration matrix for the GC samples sourced from TAGA-STAD. Subsequently, immune cell types that displayed significant differences between the two risk categories were identified. Utilizing R ggplot2, group comparison maps were then constructed to visually represent these differences. After the preliminary assessment, the Spearman algorithm was utilized to examine the link among immune cells, with R pheatmap utilized for visual depiction of these correlations via a heatmap. Additionally, to assess whether any correlation existed between the model genes and the immune cells, the Spearman algorithm was utilized. The acquired data were visualized via a correlation bubble plot that was established using R ggplot2.

### 2.12 Immunogenicity score analysis

Immunogenicity denotes the capacity of of an antigen or its specific epitopes to engage with the antigen recognition receptors found on T cells and B cells, thereby triggering either humoral or cell-mediated immune responses. The application of machine learning allows for the estimation and quantification of immunogenicity. The Cancer Immunome Atlas (TCIA) database (36) (https://tcia.at/home) offers Immunogenicity scores (IPS) for twenty distinct types of cancer, functioning as an important indicator of reactivity to CTLA-4 and PD-1. Leveraging this resource, the IPS data of GC samples were retrieved from the TCIA database, specifically pertaining to the TCGA-STAD dataset. A comparative assessment of IPS between the two risk groups was conducted by R ggplot2. The variation in IPS was analyzed.

### 2.13 Gastric adenocarcinoma tissue and RT-qPCR

The remaining postoperative specimens were collected from patients diagnosed with gastric adenocarcinoma via preoperative gastroscopy and confirmed to have no distant metastasis. If the pathological examination criteria were satisfied, we collected 5g of cancer tissue and 5g of adjacent tissue from at least 5cm away, resulting in six pairs of fresh gastric adenocarcinoma and adjacent tissue specimens. This study was conducted in accordance with the ethical principles outlined in the Declaration of Helsinki, as established by the World Medical Association. Informed consent was obtained from all participants involved in the study. Total RNA was isolated from the tissue homogenates utilizing TRIzol reagent (Invitrogen, Carlsbad, CA, USA). We performed reverse transcription according to the manufacturer’s guidelines (Takara, Jiangsu, China). The SYBR Green technique (Vazyme, Jiangsu, China) was utilized to further determine the expression levels of the target genes. To analyze the results, we used the QuantStudioTM 5 Real-Time PCR machine (Applied Biosystems, USA).

To calculate the data, the cyclic threshold (CT) (2^−DDCT^) approach was applied. Every sample underwent three assays. By employing the comparative CT approach, the expression level was normalized to that of β-actin. The primers utilized in this study are presented in **Supplementary Table S10**. We statistically analyzed the experiment data using a paired sample t-test.

### 2.14 Statistical analysis

R software (v 4.3.0) was employed for processing and analyzing the data. Statistical significance for continuous variables between the two groups was evaluated using an independent Student’s t-test, unless indicated otherwise. This approach is particularly suitable for normally distributed variables and ensures robust comparisons between groups. In cases where variables exhibited non-normal distribution, the variation between them was examined through the Mann-Whitney U Test approach (Wilcoxon Rank Sum Test). For comparison involving three or more groups, the Kruskal-Wallis test was utilized. Spearman correlation analysis was used to compute the correlation coefficient between different molecules. All statistical analyses employed two-sided *P* values unless otherwise specified, with a P value of less than 0.05 considered to signify statistical significance.

## 3 Results

### 3.1 Technology Roadmap and Standardization of Gastric Cancer Dataset

**Figure 1:**
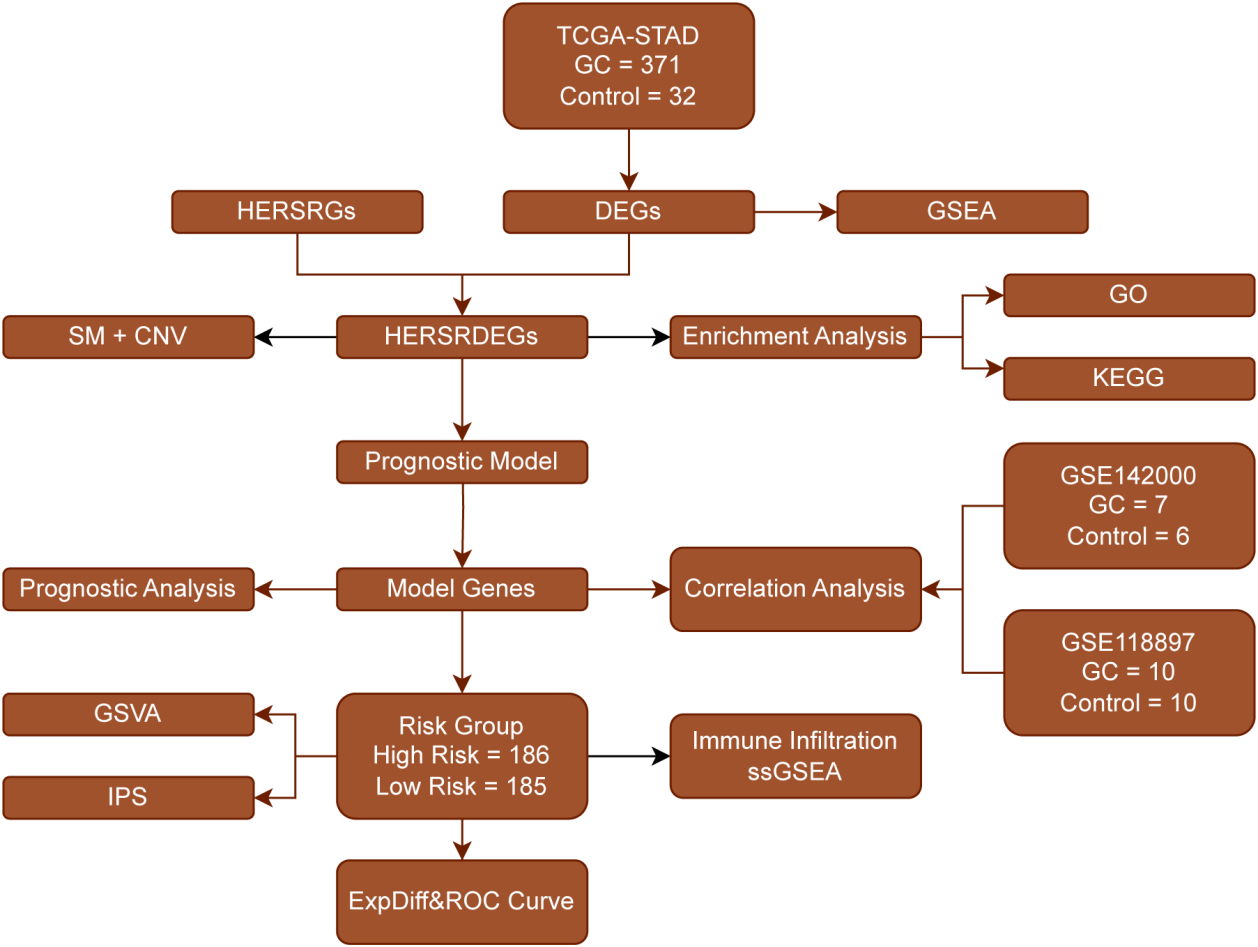
Flow Chart for the Comprehensive Analysis of HERSRDEGs TCGA, The Cancer Genome Atlas; STAD, Stomach Adenocarcinoma; GC, Gastric Cancer; DEGs, Differentially Expressed Genes; HERSRGs, hypoxia&ER Stress-Related Genes; HERSRDEGs, hypoxia&ER Stress-Related Differentially Expressed Genes; GSEA, Gene Set Enrichment Analysis; SM, Somatic Mutation; CNV, Copy Number Variations; GO, Gene Ontology; KEGG, Kyoto Encyclopedia of Genes and Genomes; GSVA, Gene Set Variation Analysis; IPS, Immunophenoscore; ssGSEA, Single-Sample Gene-Set Enrichment Analysis; ROC, Receiver Operating Characteristic.

First, we collected 371 GC samples and 32 control samples from the TCGA-STAD database. Subsequently, we determined the genes that are differentially expressed (DEGs) via an analysis of gene expression. Next, we functionally annotated the DEGs using GSEA, which included GO and KEGG pathway enrichment. Meanwhile, we identified a set of prognostically relevant genes (HERSRGs) and further selected differentially expressed genes (HERSRDEGs) significantly associated with prognosis by combining information on somatic mutation (SM) and copy number variation (CNV). Following the execution of functional enrichment analysis on the HERSRDEGs, we developed a prognostic model utilizing these genes, subsequently classifying patients into high-risk and low-risk categories. Next, we conducted correlation analysis on the modeled genes to assess their performance in independent datasets (GSE142000 and GSE118897). We also evaluated the functional status of the risk groups through GSVA and IPS, and investigated immune cell infiltration using ssGSEA. Finally, we calculated expression differences and plotted ROC curves to validate the reliability and prognostic predictive power of the model (**Figure 1**).

**Figure 2:**
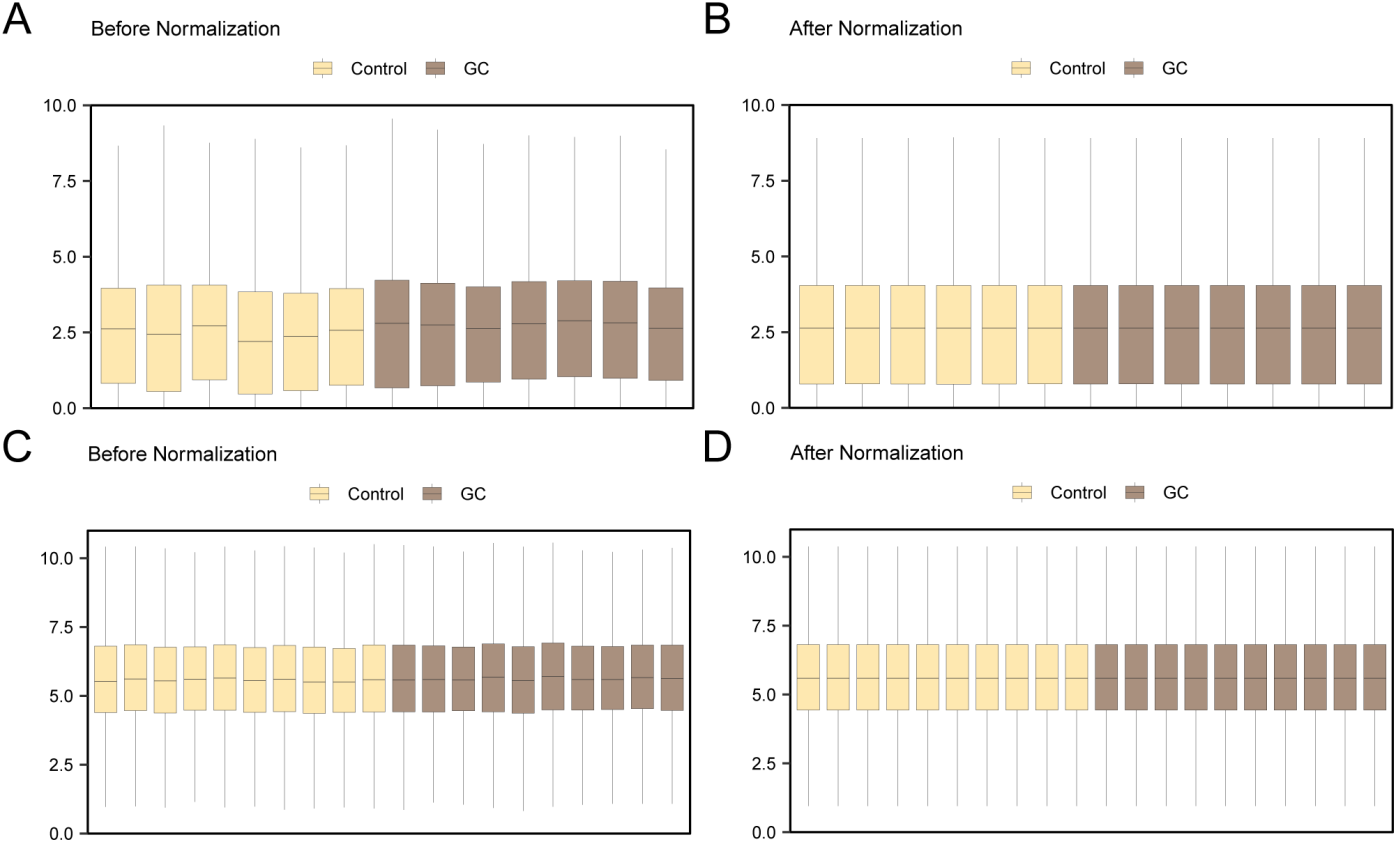
Batch Effects Removal of GSE142000 and GSE118897. **(A)** Distribution boxplot of the dataset GSE142000 prior to normalization. **(B)** Distribution boxplot of the normalized dataset GSE142000. **(C)** Distribution boxplot of dataset GSE118897 prior to normalization. **(D)** Distribution boxplot of the normalized dataset GSE118897. Yellow signifies the controls and brown signifies theGC group. GC, Gastric Cancer.

Initially, the datasets GSE142000 and GSE118897 underwent standardization, annotation of probes, and normalization via the R package limma. Following this, boxplots illustrating the distribution of expression values for GSE142000 (**Figures 2A, B**) and GSE118897 (**Figures 2C, D**) were created to evaluate the data both prior to and following normalization.

### 3.2 Gastric cancer-related hypoxia & endoplasmic reticulum stress-related differentially expressed genes

**Figure 3:**
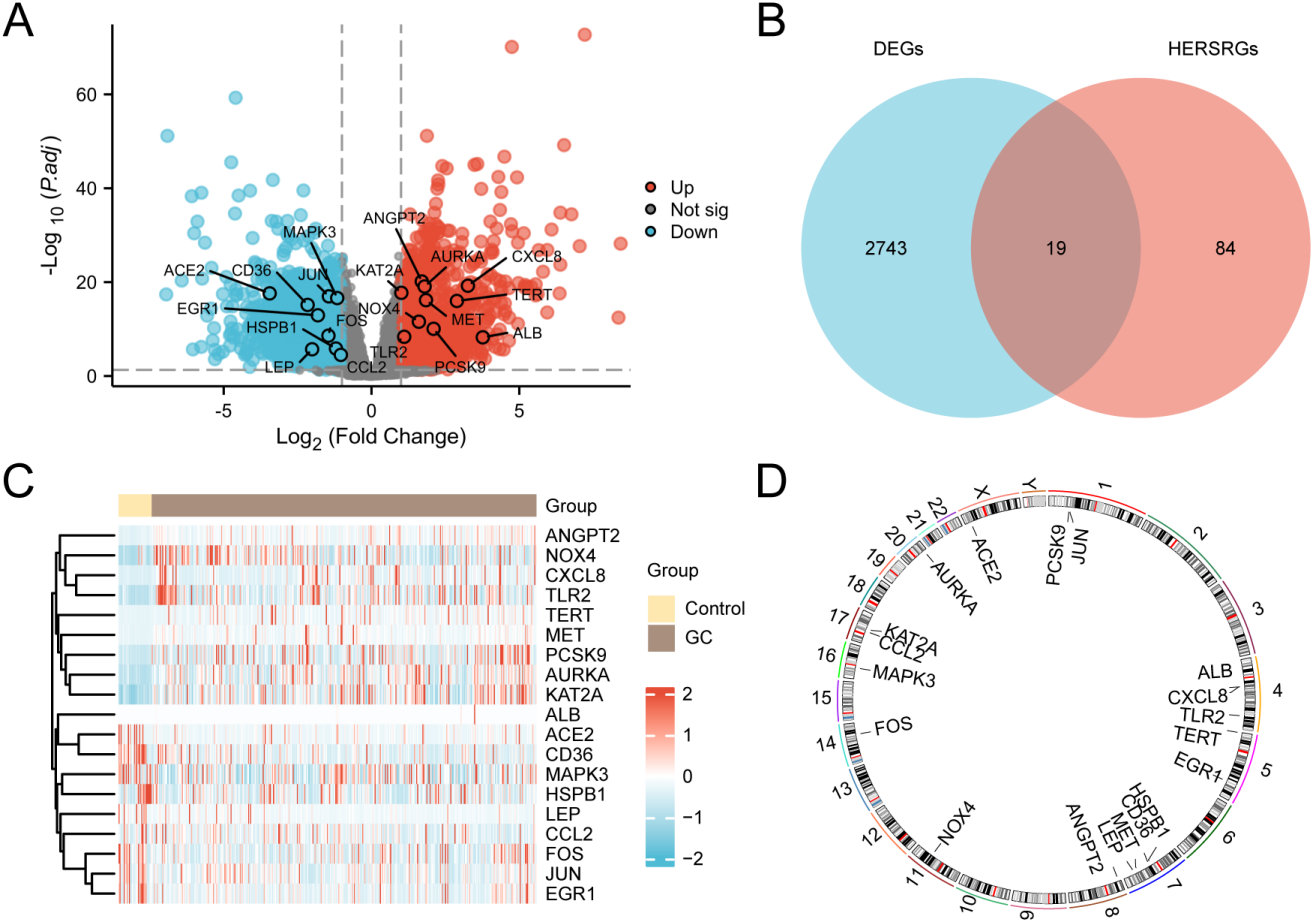
Differential Gene Expression Analysis. **(A)** Volcano plot of differentially expressed genes (DEGs) analysis between gastric cancer (GC) group and control group in the GC dataset (TCGA-STAD). **(B)** DEGs and hypoxia & endoplasmic reticulum stress-related genes (HERSRGs) Venn diagram in TCGA-STAD. **(C)** Heat map of hypoxia & ER stress-related DEGs (HERSRDEGs) in TCGA-STAD. **(D)** Chromosomal mapping of HERSRDEGs. TCGA, The Cancer Genome Atlas; STAD, Stomach Adenocarcinoma; GC, Gastric Cancer; DEGs, Differentially Expressed Genes; HERSRGs, hypoxia & ER Stress-Related Genes; HERSRDEGs, hypoxia & ER Stress-Related Differentially Expressed Genes. In the heat map group, yellow serves as the control group, and brown denotes the GC group. In the heat map, red denotes elevated expression, and blue represents reduced expression.

TCGA-STAD was divided into GC and control categories. To examine the differences in gene expression levels between these two groups, the R package DESeq2 was employed to conduct a differential analysis of the TCGA-STAD data. This analysis identified 2762 DEGs that satisfied the criteria of |logFC| > 1 and adj. P < 0.05. Among these, 1279 genes exhibited upregulated expression (logFC > 1 and adj.P < 0.05), while 1483 genes exhibited downregulated expression (logFC < -1 and adj.P < 0.05). A volcano plot was established based on these differential analysis outcomes (**Figure 3A**).

The 2762 genes obtained through differential expression analysis were integrated with the above 103 HERSRGs, and 19 HERSRDEGs were finally identified after intersection, as shown in the Venn diagram (**Figure 3B**). The identified HERSRDEGs must satisfy the following criteria: 1) They must belong to both the hypoxia-related and ER stress-related gene sets; 2) Their expression must show significant changes in the TCGA-STAD dataset, with |logFC| > 1 and adj.P < 0.05. A total of 19 HERSRDEGs were identified, namely *ANGPT2, CXCL8, AURKA, KAT2A, ACE2, JUN, MAPK3, MET, TERT, CD36, EGR1, NOX4, PCSK9, FOS, TLR2, ALB, HSPB1, LEP, CCL2*.

Following this intersection, the expression differences of HERSRDEGs in TCGA-STAD were analyzed, and a heatmap was constructed utilizing R pheatmap to visualize the analysis results (**Figure 3C**). Finally, the locations of 19 DEGs related to HERSRDEGs on human chromosomes were analyzed by the R ‘RCircos’, resulting in the generation of a chromosome localization map (**Figure 3D**). Chromosome mapping showed that more HERSRDEGs were located on chromosome 7, including HSPB1, CD36, MET, and LEP.

### 3.3 Somatic mutation, copy number variation analysis

**Figure 4:**
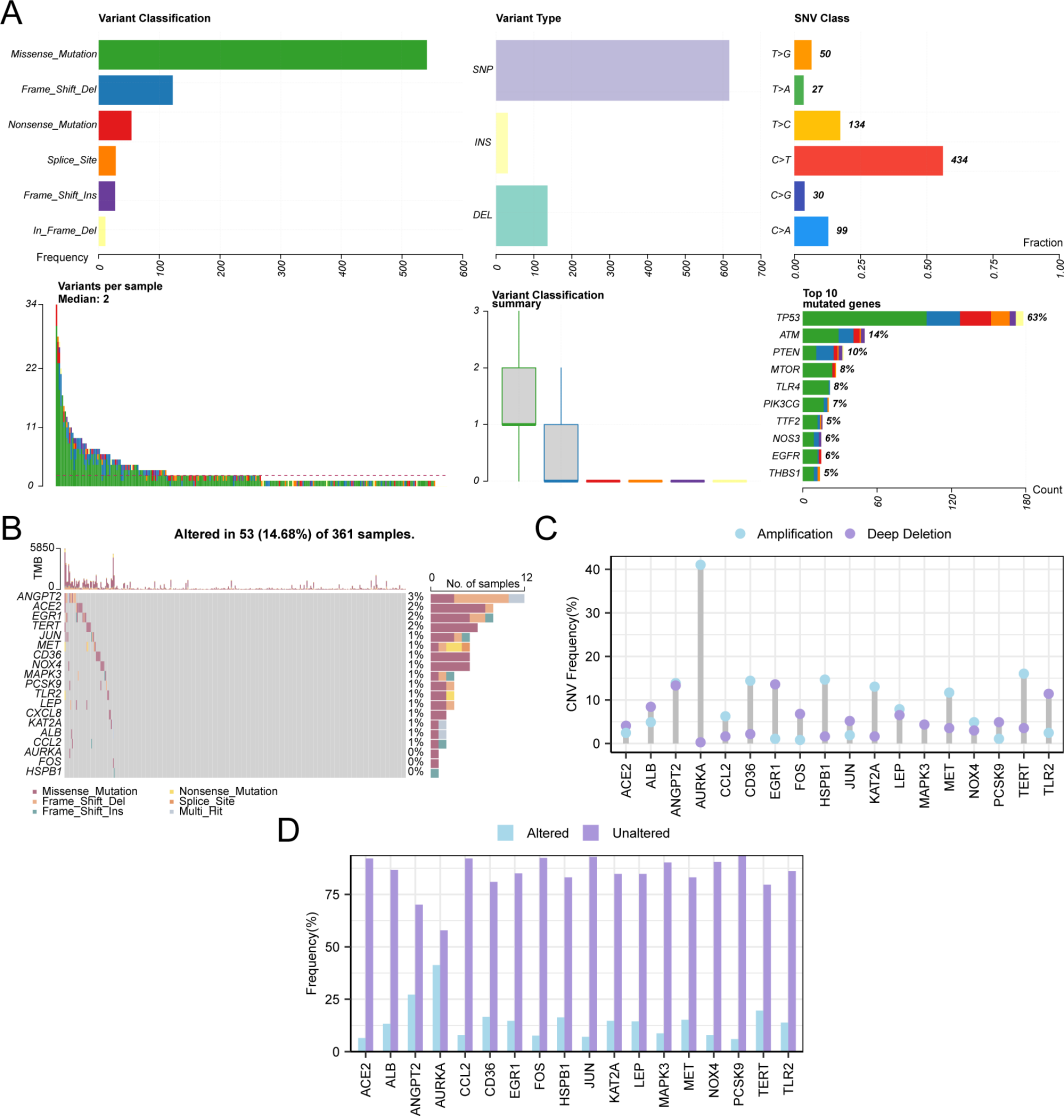
CNV and Somatic Mutation Analysis. **(A)** Presentation of somatic mutations (SM) of hypoxia & endoplasmic reticulum stress-related genes (HERSRGs) in gastric cancer (GC) samples from the Gastric Cancer dataset (TCGA-STAD). **(B)** Presentation of SM of hypoxia & endoplasmic reticulum stress-related differential genes (HERSRDEGs) in GC samples from TCGA-STAD. **(C, D)** HERSRDEGs with copy number variations (CNV) are shown in GC samples from TCGA-STAD. TCGA, The Cancer Genome Atlas; STAD, Stomach Adenocarcinoma; GC, Gastric Cancer; HERSRGs, Hypoxia & Endoplasmic Reticulum Stress-Related Genes; HERSRDEGs, hypoxia & ER Stress-Related Differentially Expressed Genes; SM, Somatic Mutation; CNV, Copy Number Variations; SNP, Single Nucleotide Polymorphism; SNV, Single Nucleotide Variant.

Initially, the analysis of SM within 103 HERSRGs in GC samples from the Gastric Cancer Dataset (TCGA-STAD) was conducted, followed by visualization using the R package maftools (**Figure 4A**). The findings revealed six predominant SM types within HERSRGs, with missense mutations being predominant. Notably, the predominant mutation type among the 103 HERSRGs in GC samples was Single Nucleotide Polymorphism (SNP), with C to T mutation emerging as the most prevalent Single Nucleotide Variant (SNV) in GC samples. Subsequently, an analysis of the SM status of 19 HERSRDEGs in GC samples was performed, followed by ranking them based on mutation frequency from high to low and visualizing these genes (**Figure 4B**). The results indicated that ANGPT2 exhibited the highest mutation rate of 3%.

For the examination of CNV among 19 HERSRDEGs in GC samples sourced from TCGA-STAD, CNV data of GC samples from the same dataset were obtained and merged. Utilizing GISTIC2.0 analysis, CNV was identified in 18 out of the 19 HERSRDEGs within GC samples. Subsequently, the mutation status of these 18 genes exhibiting CNV was depicted (**Figures 4C, D**), comprising: *ACE2, ALB, ANGPT2, AURKA, CCL2, CD36, EGR1, FOS, HSPB1, JUN, KAT2A, LEP, MAPK3, MET, NOX4, PCSK9, TERT, TLR2.*

### 3.4 Correlation analysis of differentially expressed genes associated with hypoxia and endoplasmic reticulum stress

**Figure 5:**
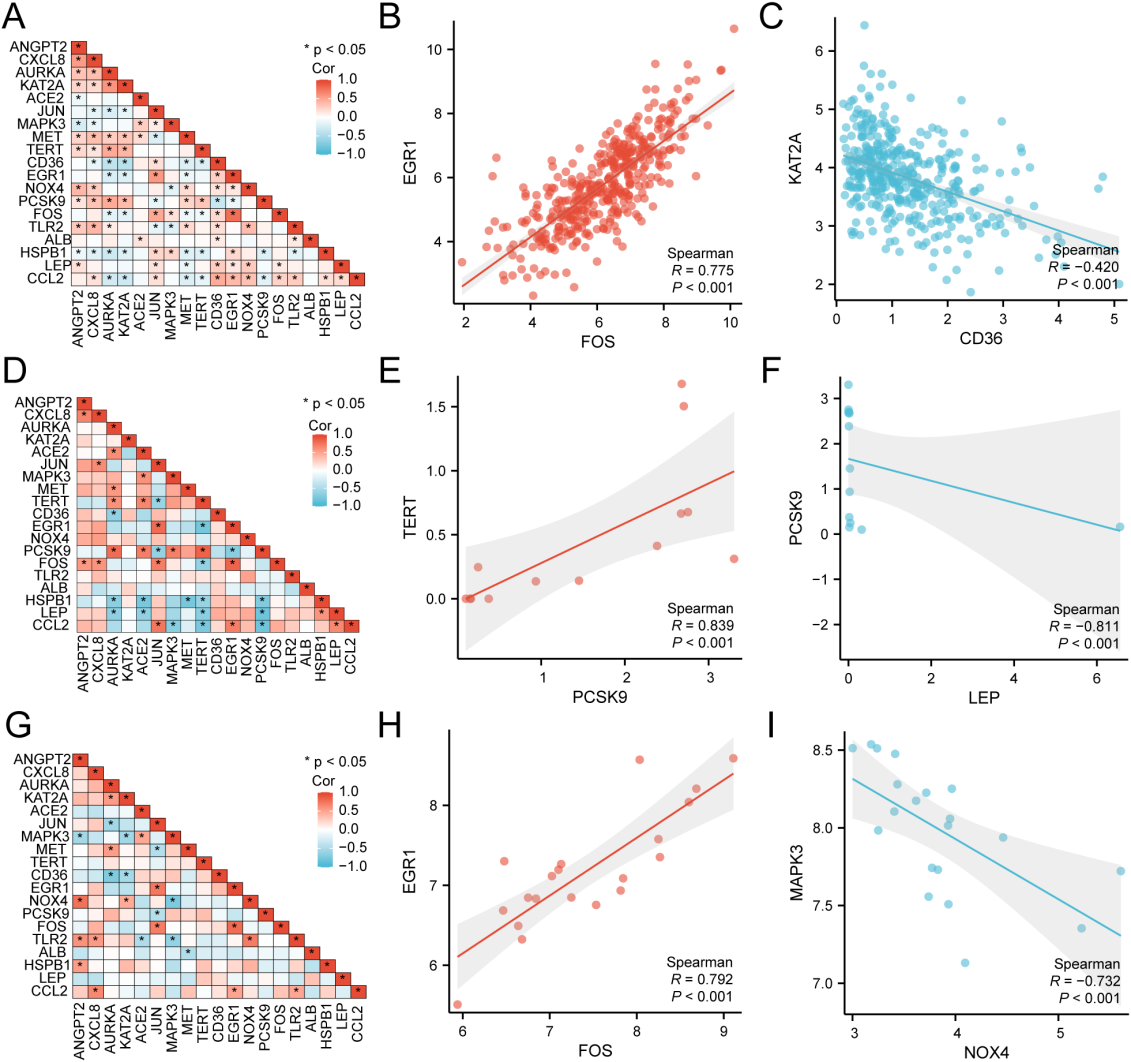
Correlation Analysis of HERSRDEGs **(A)** Correlation heat map of 19 hypoxia & endoplasmic reticulum stress-related differentially expressed genes (HERSRDEGs) in gastric cancer dataset (TCGA-STAD). B-C. Scatter plot of HERSRDEGs EGR1 and FOS **(B)**, KAT2A, and CD36 **(C)** in TCGA-STAD. **(D)** Correlation heat map of 19 HERSRDEGs in dataset GSE142000. **(E, F)** Scatter plot of HERSRDEGs TERT and PCSK9 **(E)**, PCSK9, and LEP **(F)** in dataset GSE142000. **(G)** Correlation heat map of 19 HERSRDEGs in dataset GSE118897. H-I. Scatter plot of HERSRDEGs EGR1 and FOS **(H)**, MAPK3, and NOX4 **(I)** in dataset GSE118897. TCGA, The Cancer Genome Atlas; STAD, Stomach Adenocarcinoma; HERSRDEGs, Hypoxia & Endoplasmic Reticulum Stress-Related Differentially Expressed Genes. The absolute value of the correlation coefficient (r-value) below 0.3 signifies weak or no correlation, while values between 0.3 and 0.5 signify weak correlation, and those between 0.5 and 0.8 denote moderate correlation. Values above 0.8 signify a strong correlation. Red depicts a positive correlation, and blue depicts a negative correlation. A *P*-value < 0.05 was deemed to represent statistical significance.

Utilizing the comprehensive expression matrix of 19 HERSRDEGs from TCGA-STAD, correlation analysis was conducted, followed by visualization via a correlation heat map (**Figure 5A**). Subsequently, correlation scatter plots were employed to illustrate the results of the correlation analysis concerning the top positively and negatively correlated genes identified in the heat map (**Figures 5B, C**). Notably, *EGR1* and *FOS* exhibited a significant positive correlation within TCGA-STAD (P value < 0.05, r value = 0.775), while *KAT2A* and *CD36* demonstrated a significant negative correlation (P value < 0.05, r value = -0.420).

Subsequently, utilizing the complete expression matrix of 19 HERSRDEGs in dataset GSE142000, correlation analysis was conducted, followed by visualization through a correlation heat map (**Figure 5D**). Further, correlation scatter plots were employed to present the results of correlation analysis between the top1 positively and negatively correlated genes identified in the correlation heat map (**Figures 5E, F**). Remarkably, a significant positive link was noted between *TERT* and *PCSK9* in GSE142000 (P < 0.05, r value = 0.839), while a considerable negative link was found between *PCSK9* and *LEP* in the same dataset (P value < 0.05, r value = -0.811).

Lastly, utilizing the complete expression matrix of 19 HERSRDEGs in dataset GSE118897, correlation analysis was conducted, accompanied by visualization through a correlation heat map (**Figure 5G**). Subsequent to this, correlation scatter plots were employed to exhibit the findings of correlation analysis between the top1 positively and negatively correlated genes identified in the heat map (**Figures 5H, I**). Notably, a considerable positive link was observed between *EGR1* and *FOS* in GSE118897 (P < 0.05, r value = 0.792). Additionally, *MAPK3* and *NOX4* showed a significant negative correlation in the same dataset (P value < 0.05, r value = -0.732).

### 3.5 Gene ontology and pathway (KEGG) enrichment analysis

**Figure 6:**
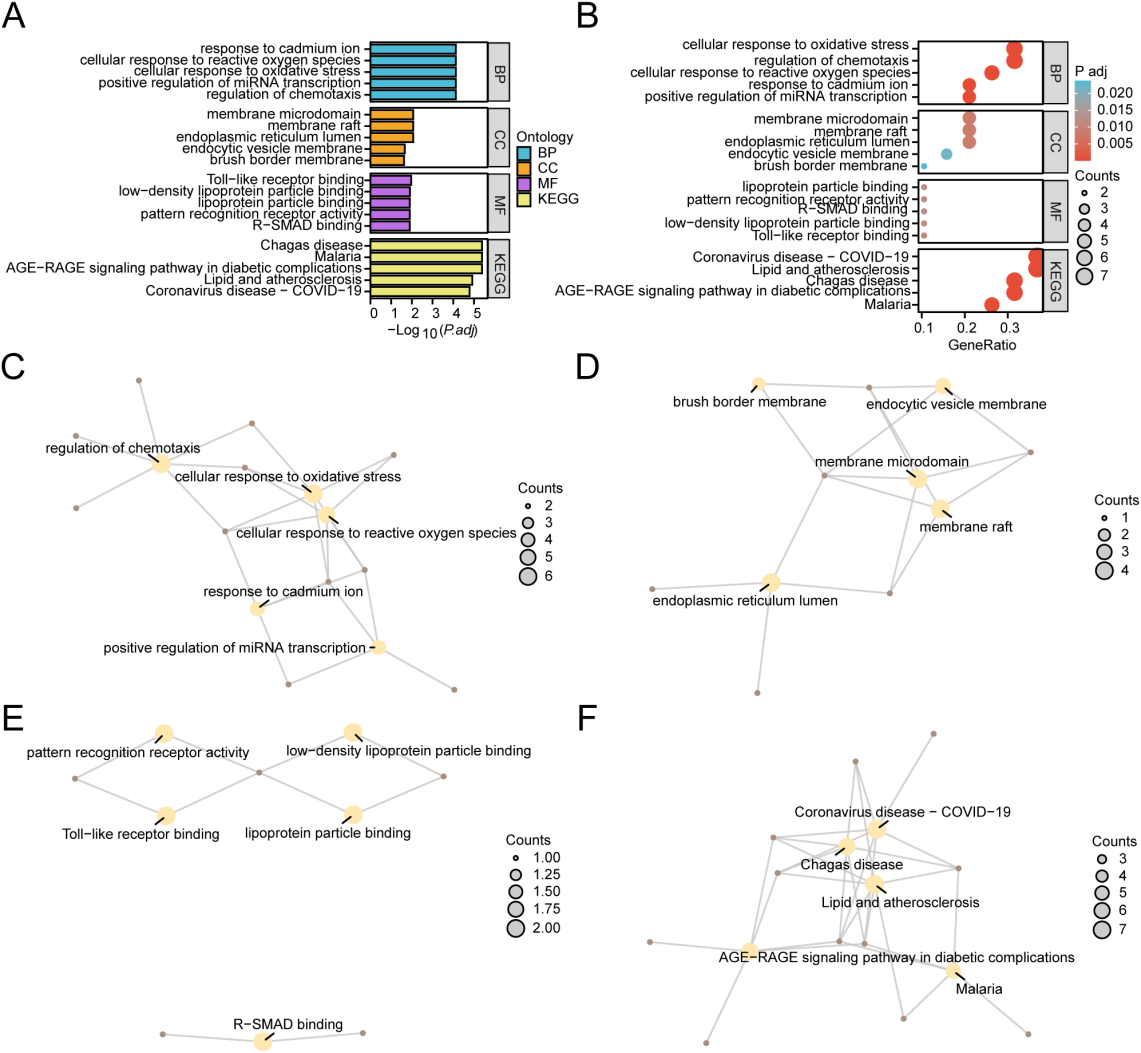
GO and KEGG Enrichment Analyses for HERSRDEGs. **(A, B)** Gene ontology (GO) and pathway (KEGG) enrichment analyses results of hypoxia & endoplasmic reticulum stress-related differentially expressed genes (HERSRDEGs) Bar graph **(A)** and bubble plot **(B)** illustrates biological process (BP), cellular component (CC), molecular function (MF) and biological pathway (KEGG). GO terms and KEGG terms are shown on the ordinate. **(C–F)** GO and KEGG outcomes of HERSRDEGs: BP **(C)**, CC **(D)**, MF **(E)**, and KEGG **(F)**. Yellow nodes denote items, brown nodes denote molecules, and lines denote the link between items and molecules. HERSRDEGs, Hypoxia & Endoplasmic Reticulum Stress-Related Differentially Expressed Genes; GO, Gene Ontology; KEGG, Kyoto Encyclopedia of Genes and Genomes; BP, Biological Process; CC, Cell Component; MF, Molecular Function. The bubble size in the bubble plot depicts the gene count, and the color of the bubble depicts the size of the adj. *P*-value, the redder the color, the smaller the adj. *P*-value, while the bluer color corresponds to a larger adj. *P*-value. The screening criteria for GO and KEGG enrichment analysis were adj.*P* < 0.05 and FDR value (q value) < 0.25. The *P* value correction method utilized was Benjamini-Hochberg (BH).

GO and KEGG were employed to delve deeper into the link between BP, CC, MF, and biological pathway (KEGG) of the 19 HERSRDEGs associated with GC. The specific outcomes are detailed in **Supplementary Table S9**. The analysis highlighted that the 19 HERSRDEGs were primarily enriched in several key BPs, including the regulation of chemotaxis, positive regulation of miRNA transcription, cellular responses to oxidative stress, reactive oxygen species, and cadmium ion. Regarding CC, enrichment was observed in the endoplasmic reticulum lumen, membrane raft, membrane microdomain, endocytic vesicle, and brush border cells. Furthermore, concerning molecular functions (MF), the genes exhibited notable enrichment in activities such as Toll-like receptor binding, binding to low-density lipoprotein particles, interaction with R-SMAD, pattern recognition receptor activity, and lipoprotein particle binding. Additionally, enrichment was detected in the AGE-RAGE pathway in diabetic complications, as well as pathways related to Malaria, Chagas disease, Lipid and atherosclerosis, and Coronavirus disease (COVID-19) within KEGG. The outcomes of GO and KEGG enrichment analyses were visually presented utilizing bar plots (**Figure 6A**) and bubble plots (**Figure 6B**).

Concurrently, a network diagram illustrating BP, CC, MF, and biological pathway (KEGG) was generated based on GO and KEGG analysis (**Figures 6C–F**). The diagram depicts lines connecting the associated molecules and provides annotations for the relevant entries. Larger nodes indicate higher molecule counts within the entries. Notably, the results revealed a greater enrichment of genes in pathways related to Lipid and atherosclerosis, as well as COVID-19 within the KEGG pathways.

### 3.6 Gene set Enrichment analysis

**Figure 7:**
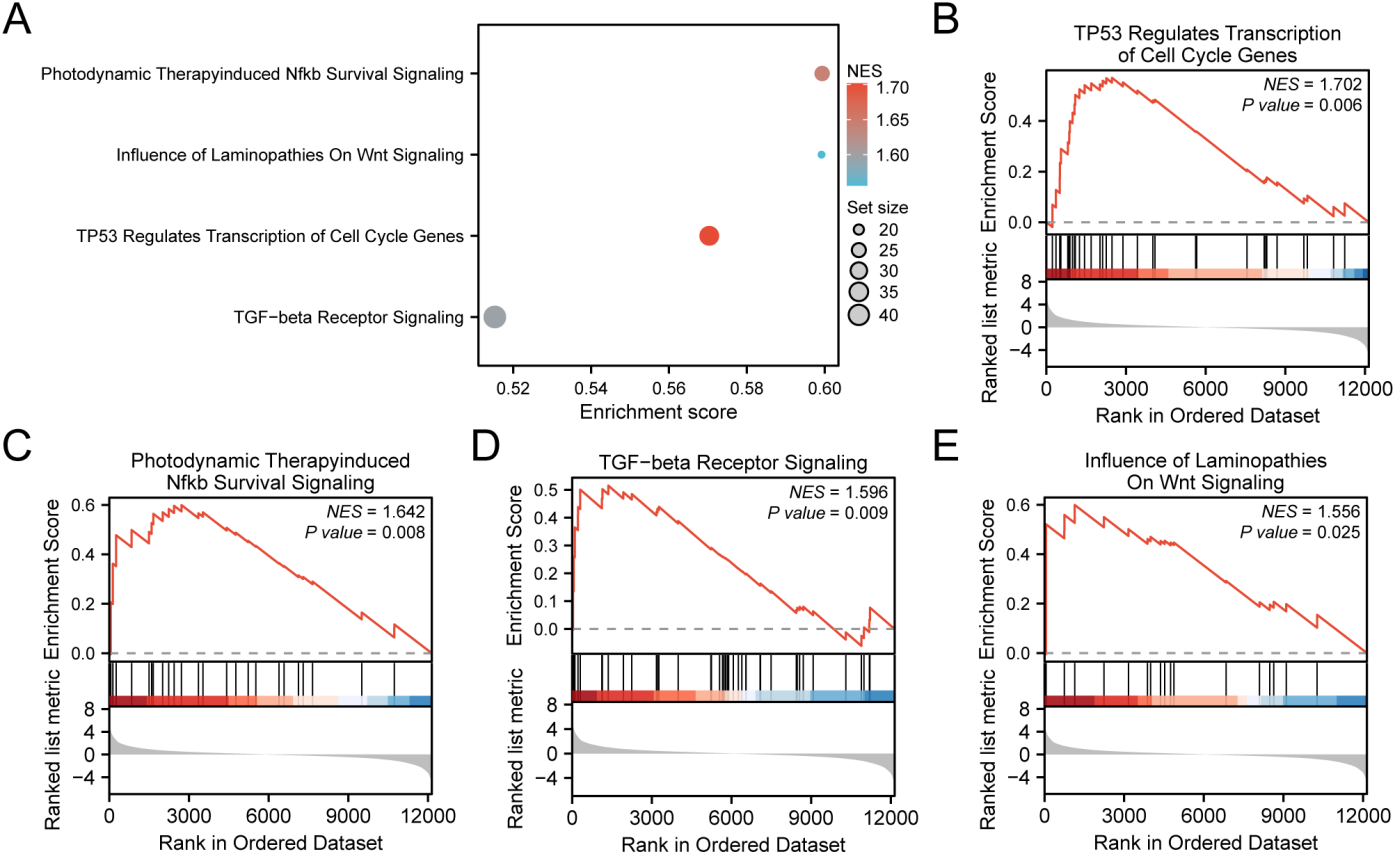
GSEA for TCGA-STAD. **(A)** Bubble plot presentation of 4 biological functions from gene set enrichment analysis (GSEA) of gastric cancer dataset (TCGA-STAD). B-E. GSEA showed that all genes exhibited considerable enrichment in TP53 Regulates Transcription of Cell Cycle Genes **(B)**. Photodynamic Therapy induced Nfkb Survival Signaling **(C)**, TGF−beta Receptor Signaling **(D)**, and Influence of Laminopathies On Wnt Signaling **(E)**. TCGA, The Cancer Genome Atlas; STAD, Stomach Adenocarcinoma; GSEA, Gene Set Enrichment Analysis. In the bubble plot, the size of the bubble depicts the number of enriched genes, and the color of the bubble depicts the size of the NES value. The intensity of the red color signifies a higher NES value, while a bluer color indicates a lower NES value. The screening criterion of GSEA was *P* value < 0.05.

For assessing the effect of gene expression levels across TCGA-STAD on GC, GSEA was conducted. The assessment aimed to explore the association between the expression of all genes within TCGA-STAD and the BP, CC, and MF they regulate (**Figure 7A**). Detailed outcomes are illustrated in **Supplementary Table S4**. The findings demonstrated considerable enrichment of all genes in TCGA-STAD in several biological functions and signaling pathways, including TP53 Regulates Transcription of Cell Cycle Genes (**Figure 7B**), Photodynamic Therapy-induced Nfkb Survival Signaling (**Figure 7C**), TGF-beta Receptor Signaling (**Figure 7D**), Influence of Laminopathies On Wnt Signaling (**Figure 7E**), and others.

### 3.7 Development of predictive risk model for gastric cancer

**Figure 8:**
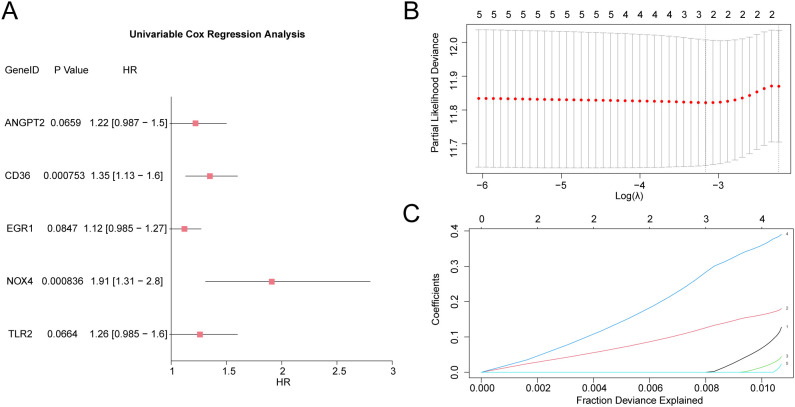
LASSO and Cox Regression Analysis **(A)** Forest Plot of 5 hypoxia & endoplasmic reticulum stress-related differentially expressed genes (HERSRDEGs) in univariate Cox regression model. **(B, C)** Plots of prognostic risk models **(B)** and variable trajectories **(C)** from the LASSO regression model. HERSRDEGs, Hypoxia & ER Stress-Related Differentially Expressed Genes; LASSO, Least Absolute Shrinkage and Selection Operator.

To develop a predictive risk model for GC, univariate Cox regression analysis was executed using 19 HERSRDEGs. Variables with a *P* value < 0.10 in univariate analysis were visualized by a Forest Plot (**Figure 8A**). The findings highlighted that five HERSRDEGs were statistically significant (*P* < 0.10) in the univariate Cox regression model. These genes included *ANGPT2, CD36, EGR1, NOX4, TLR2.*

To further evaluate the prognostic value of these HERSRDEGs, a LASSO regression analysis was executed, and a LASSO regression model was constructed. The LASSO regression model diagram (**Figure 8B**) and LASSO variable trajectory diagram (**Figure 8C**) were utilized for visualization. The results revealed that the LASSO regression model comprised three significant genes: *ANGPT2*, *CD36*, and *NOX4*. The RiskScore was computed utilizing the following formula:


RiskScore = ANGPT2*(0.002) + CD36*(0.133) + NOX4*(0.3)

### 3.8 Prognostic analysis of gastric cancer prognostic risk model

**Figure 9:**
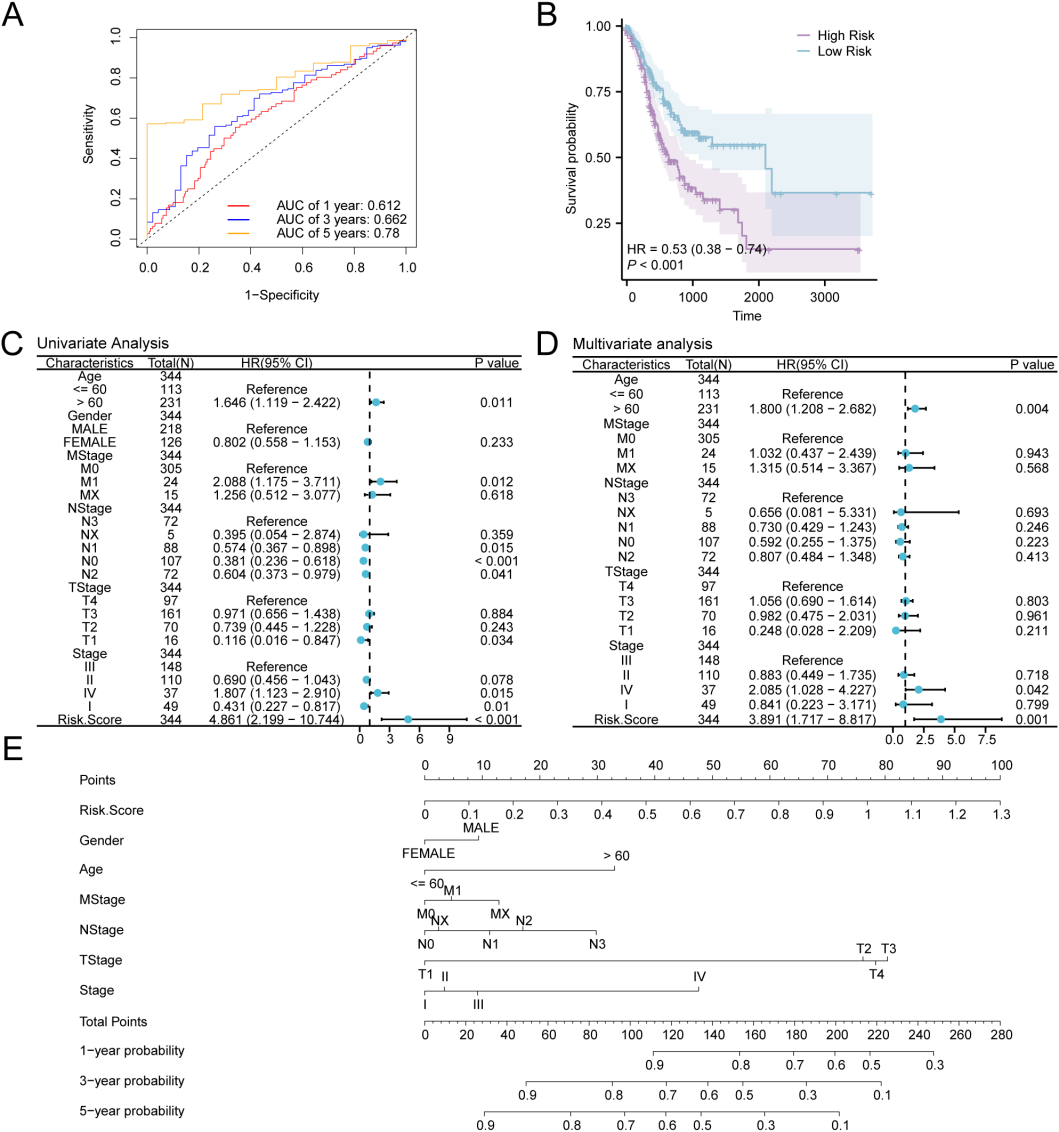
Prognostic Analysis. **(A)** Time-dependent ROC curves of gastric cancer (GC) samples in the GC dataset (TCGA-STAD). **(B)** Prognostic KM curves between low- and high-risk groups and overall survival (OS) of GC samples. **(C, D)** Forest Plot of RiskScore and clinical data in univariate **(C)** and multivariate Cox regression model **(D)**. **(E)** Nomogram of RiskScore and clinical information in Cox regression models (univariate and multivariate). TCGA, The Cancer Genome Atlas; STAD, Stomach Adenocarcinoma; GC, Gastric Cancer; OS, Overall Survival; KM, Kaplan-Meier; ROC, Receiver Operating Characteristic Curve; AUC, Area Under the Curve.

We validated the model’s performance with time-dependent ROC curves (**Figure 9A**), showing its ability to predict survival at various time points (1, 3, and 5 years). We evaluate the model’s prediction accuracy using the AUC value. The model demonstrated strong accuracy for the 5-year prognosis (AUC > 0.7), indicating that our risk score model is highly effective for long-term predictions. Additionally, prognostic KM curve analysis was performed in accordance with the median grouping of OS of GC samples in the RiskScore combined GC dataset TCGA-STAD (**Figure 9B**). The findings demonstrated a significant statistical difference in overall survival (OS) between the high-risk and low-risk cohorts, as well as the GC sample in the TCGA-STAD (P-value < 0.001). Subsequently, univariate Cox analysis was carried out utilizing the median RiskScore grouping combined with clinical information and OS of GC samples. The screening of variables exhibiting a *P*-value < 0.10 was conducted for multivariate Cox analysis. The outcomes of both Cox regression analyses were visualized through forest plots (**Figures 9C, D**), which are provided in **Supplementary Table S5**. The findings from the single multivariate Cox regression analysis demonstrated that RiskScore and clinical information, encompassing age and stage, were statistically significant (*P*-value < 0.05).

For further exploration of the prognostic value of the GC risk model, a nomogram was developed as per the outcomes of Cox regression analyses (univariate and multivariate) to show the relationship between RiskScore and 6 clinical variables in GC samples (**Figure 9E**). The findings indicate that the efficacy of RiskScore in the GC prognostic risk model significantly surpasses that of other variables, while the utility of gender for the aforementioned model is significantly lower than that of other variables.

When AUC > 0.5, it suggests that the expression of the molecule is associated with promoting the occurrence of the event. Moreover, a value of AUC closer to 1 demonstrates a stronger diagnostic effect. AUC ranging from 0.5 to 0.7 denotes low accuracy, whereas values from 0.7 to 0.9 suggest moderate accuracy. A *P*-value of < 0.001 demonstrates elevated statistical significance.

**Figure 10:**
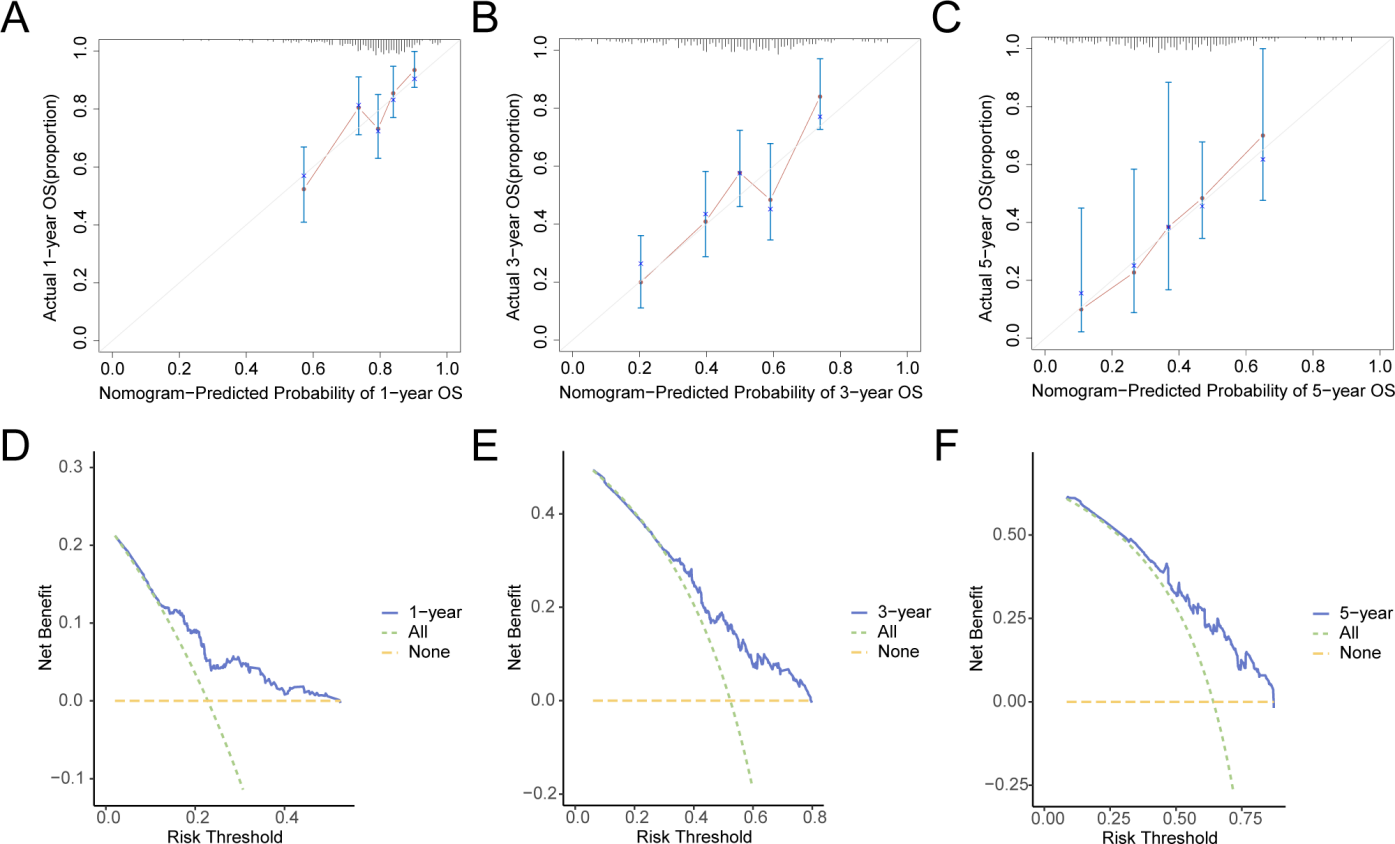
Prognostic Analysis. **(A–C)** 1-year **(A)**, 3-year **(B)**, 5-year **(C)** calibration curve of a prognostic risk model for gastric cancer (GC). D-F. 1-year **(D)**, 3-year **(E)**, and 5-year **(F)** decision curve analysis (DCA) plots of the GC prognostic risk model. GC, Gastric Cancer; DCA, Decision Curve Analysis.

Additionally, calibration analysis was carried out on the GC prognostic risk model at 1-year, 3-year, and 5-year, and a calibration curve was plotted (**Figures 10A–C**). On the calibration curve, the y-axis represents the observed survival probability derived from empirical data, whereas the x-axis reflects the survival probability as estimated by the model. The line representing different time points predicted by the model is closer to the line of the gray ideal case, signifying better predictive performance at those time specific points. The results indicated that the GC model demonstrated superior clinical predictive capability over a 5-year period. Finally, DCA was conducted to carry out a clinical utility assessment of this model at 1, 3, and 5 years (**Figures 10D–F**). The findings depicted that the clinical predictive effect of the LASSO regression model was 5-year > 3-year > 1-year.

### 3.9 Differential expression verification and ROC curve analysis between the risk groups

**Figure 11:**
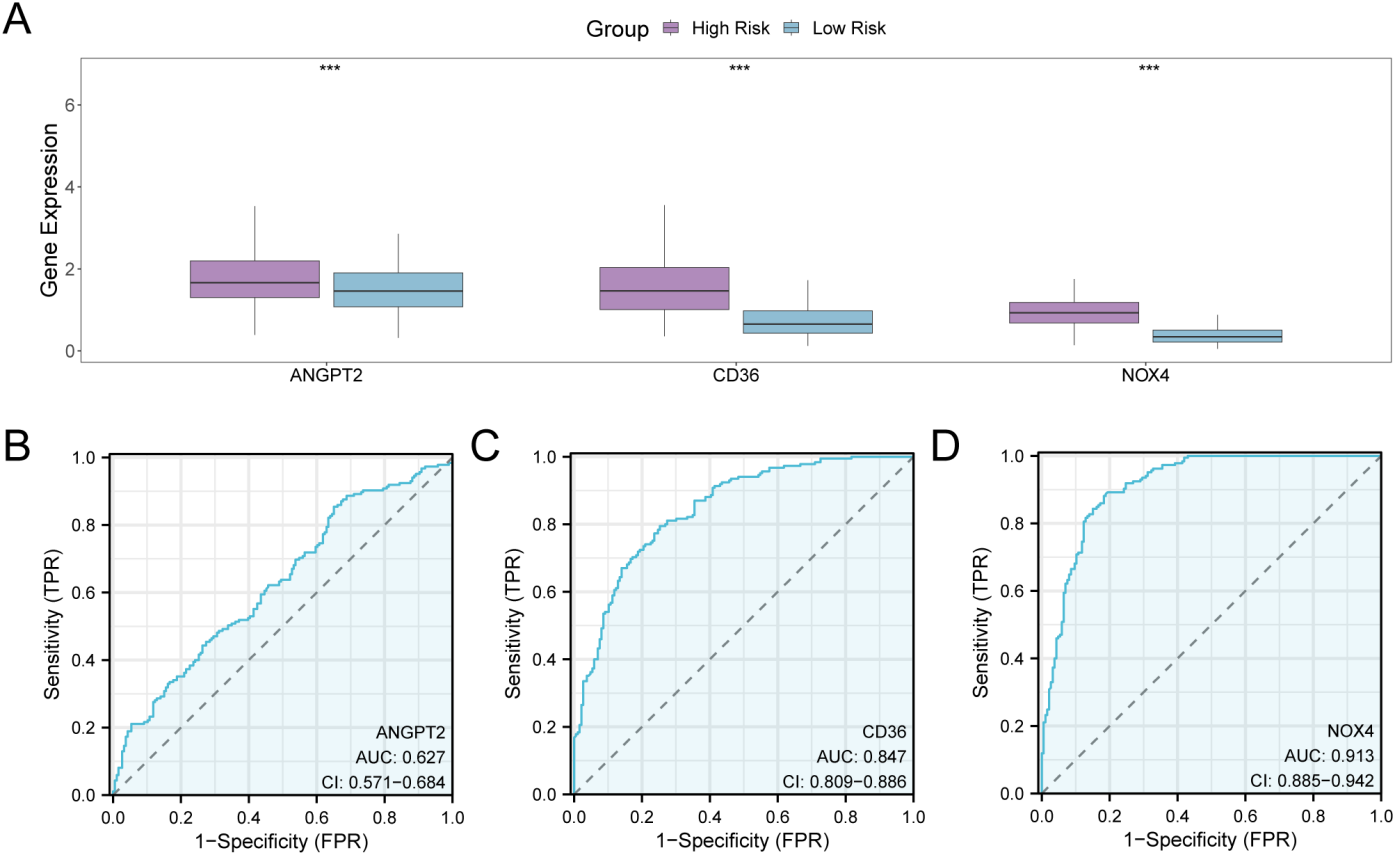
Differential Expression Validation and ROC Curve Analysis. **(A)** Group comparison plot of model genes in the high- and low-risk groups of gastric cancer (GC) samples in the GC dataset (TCGA-STAD). **(B–D)** ROC curves of model genes ANGPT2 **(B)**, CD36, **(C)**, and NOX4 **(D)** in GC samples from TCGA-STAD. TCGA, The Cancer Genome Atlas; STAD, Stomach Adenocarcinoma; GC, Gastric Cancer; ROC, Receiver Operating Characteristic; AUC, Area Under the Curve; TPR, True Positive Rate; FPR, False Positive Rate. *** denotes a P-value < 0.001, indicating considerable statistical significance. When AUC > 0.5, it indicates that the expression of the molecule is associated with promoting the occurrence of the event, with values closer to 1 indicating a better diagnostic impact. The AUC values within the range of 0.5 and 0.7 are correlated with low accuracy, those between 0.7 and 0.9 had moderate accuracy, and values greater than 0.9 depict high accuracy. The high- and low-risk groups are shown in purple and blue, respectively.

The stratification of GC samples from TCGA-STAD into high-risk and low-risk groups was carried out as per the median expression value of RiskScore derived from the GC prognostic risk model. To explore the differential expression of model genes within GC samples, a comparative analysis was performed (**Figure 11A**), This analysis revealed the variations in expression levels of three model genes across the specified groups. The differential results revealed that three model genes, namely *ANGPT2*, *CD36*, and *NOX4*, exhibited high statistical significance in expression levels between the above-mentioned risk groups (*P*-value < 0.001). Subsequently, R pROC was utilized to generate ROC curves as per the expression levels of model genes (**Figures 11B–D**). The ROC curve illustrated that the expression level of the model gene, NOX4, depicted greater accuracy (AUC > 0.9) in distinguishing high- and low-risk groups. Additionally, the expression level of CD36 depicted a moderate accuracy (0.7 < AUC < 0.9), while the expression level of *ANGPT2* showed lower accuracy (0.5 < AUC < 0.7) in this differentiation.

### 3.10 Gene-set variation analysis for the risk groups

**Figure 12:**
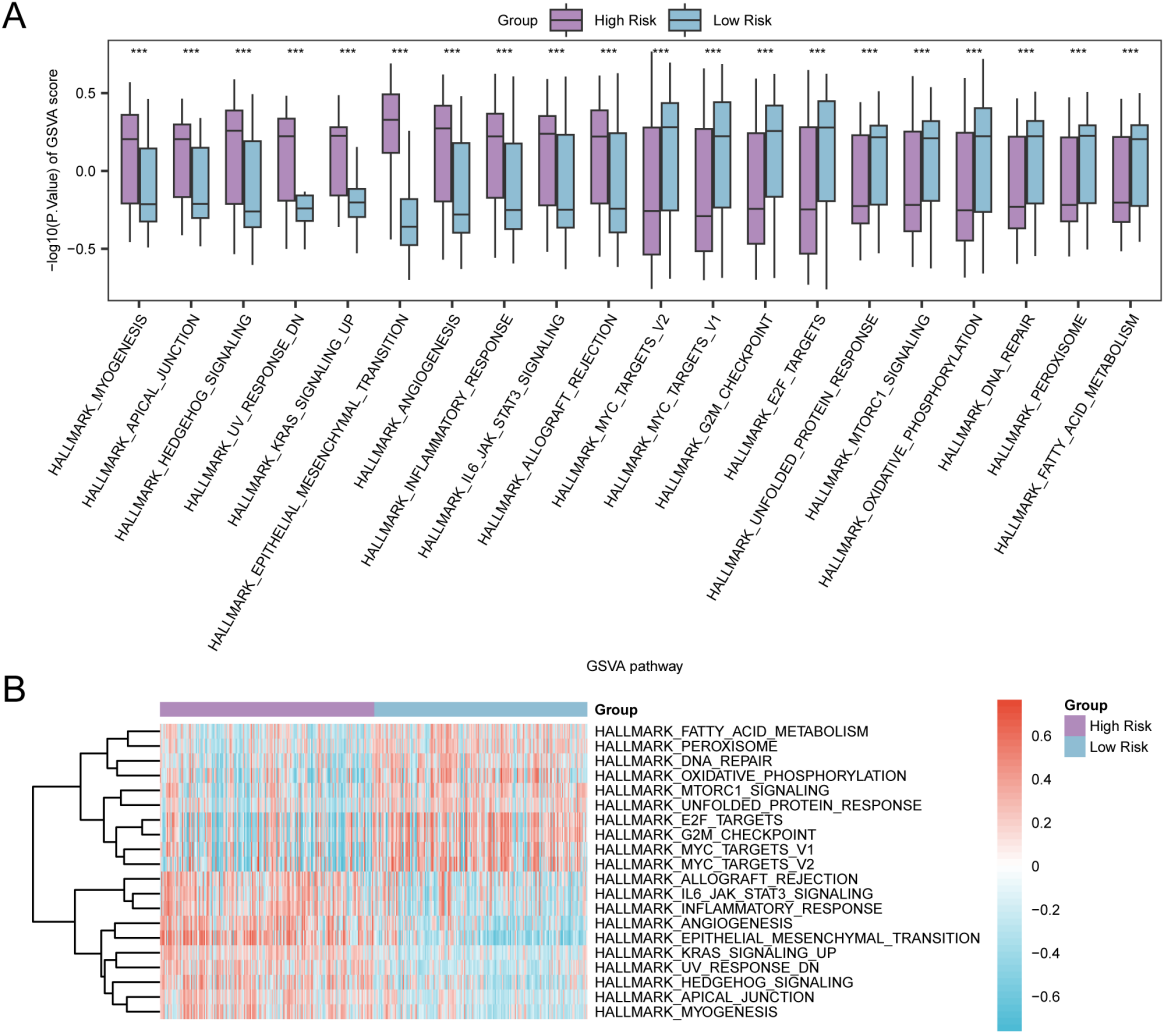
GSVA for Risk Group. **(A, B)** Group comparison plot **(A)** and heat map **(B)** of gene set variation analysis (GSVA) results between high-risk group and low-risk group in gastric cancer (GC) samples of TCGA-STAD. TCGA, the Cancer Genome Atlas; STAD, Stomach Adenocarcinoma; GC, Gastric Cancer; GSVA, Gene Set Variation Analysis. *** denotes a P-value < 0.001, suggesting considerable statistical significance. The blue color indicates the low-risk group, whereas purple signifies the high-risk group. In the heat map, red signifies high enrichment, whereas blue signifies low enrichment. The screening criteria for GSVA was set as adj.P < 0.05, with the Benjamini-Hochberg approach utilized for *P*-value correction.

GSVA was performed on the complete set of genes from the GC samples in TCGA-STAD to assess the variability of the h.all.v2023.2.Hs.symbols.GMT gene set between both risk groups. Comprehensive details pertaining to the analysis can be found in **Supplementary Table S6**. Subsequently, pathways exhibiting positive enrichment that had an adjusted p-value (adj.P) of less than 0.05 and ranked within the top 10 for logFC were identified, in addition to the top 10 pathways demonstrating negative enrichment. The findings were illustrated by conducting a group comparison (**Figure 12A**). The GSVA outcomes revealed that 20 pathways exhibited strong statistical significance (P-value < 0.001) in both risk groups of GC samples within TCGA-STAD. These pathways are listed below: FATTY ACID METABOLISM, PEROXISOME, DNA REPAIR, OXIDATIVE PHOSPHORYLATION, MTORC1 SIGNALING, UNFOLDED PROTEIN RESPONSE, E2F TARGETS, G2M CHECKPOINT, MYC TARGETS V1, MYC TARGETS V2, ALLOGRAFT REJECTION, IL6 JAK STAT3 SIGNALING, INFLAMMATORY RESPONSE, ANGIOGENESIS, EPITHELIAL MESENCHYMAL TRANSITION, KRAS SIGNALING UP, UV RESPONSE DN, HEDGEHOG SIGNALING, APICAL JUNCTION, and MYOGENESIS. Finally, on the basis of GSVA findings, a heat map was employed to examine and visualize the differential expression of 20 pathways between the two groups (high- and low-risk) in GC samples from TCGA-STAD (**Figure 12B**).

### 3.11 Immune infiltration analysis in the risk groups

**Figure 13:**
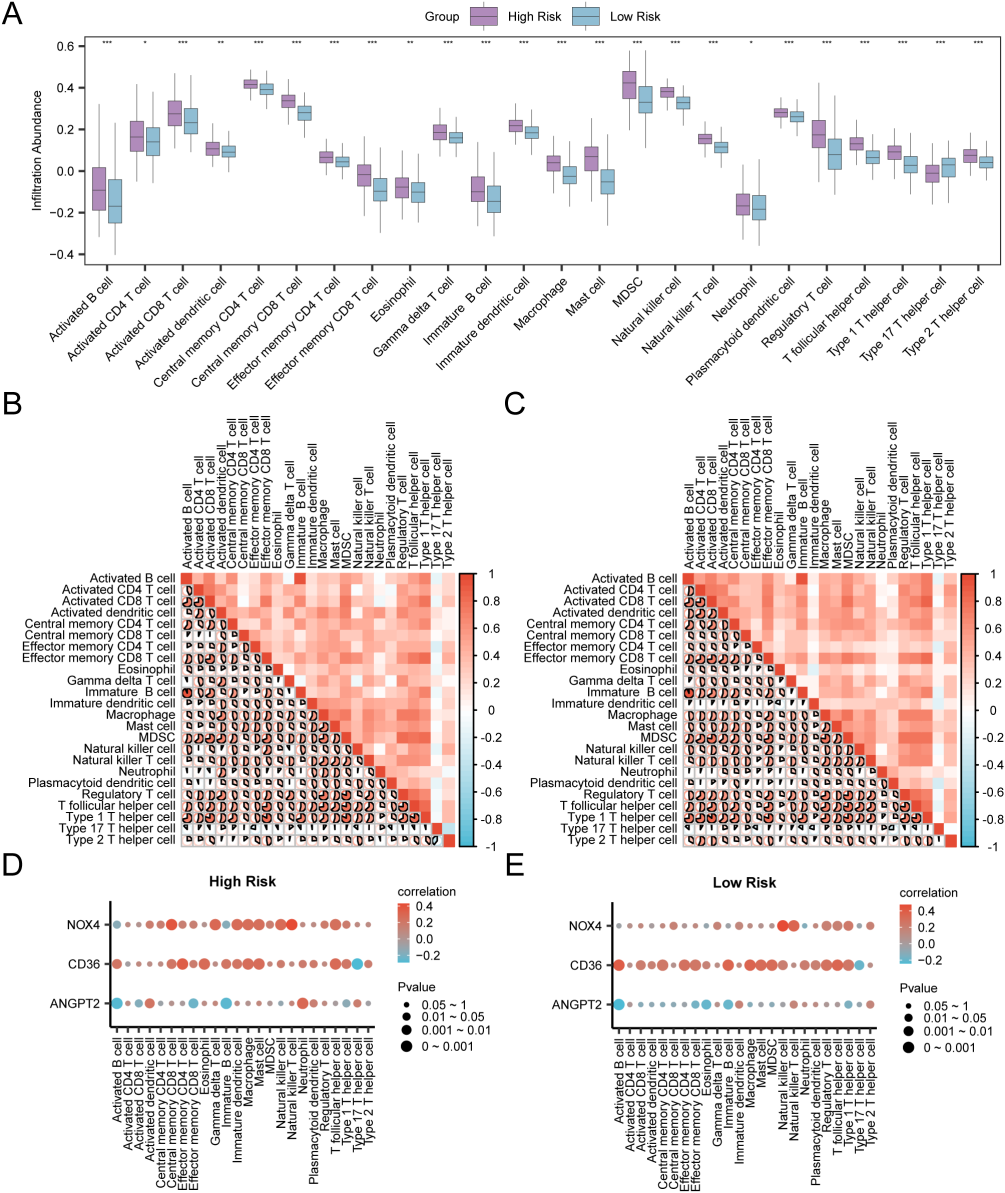
Risk Group Immune Infiltration Analysis by ssGSEA Algorithm **(A)** Comparison of the grouping of immune cells in the High Risk group and the Low Risk group of gastric cancer (GC) samples. **(B, C)** Results of correlation analysis of immune cell infiltration abundance in the High Risk group **(B)** and the Low Risk group **(C)** of gastric cancer (GC) samples are shown. **(D, E)** Bubble plot of correlation between immune cell infiltration abundance and Model Genes in High Risk **(D)** and Low Risk **(E)** groups of gastric cancer (GC). ssGSEA, single-sample Gene-Set Enrichment Analysis; GC, Gastric Cancer. * represents P value < 0.05, statistically significant; ** represents p value < 0.01, highly statistically significant; *** represents P value < 0.001 and highly statistically significant. The absolute value of correlation coefficient (r value) below 0.3 was weak or no correlation, between 0.3 and 0.5 was weak correlation, between 0.5 and 0.8 was moderate correlation, and above 0.8 was strong correlation. In the group comparison diagram, purple is the High Risk group, and blue is the Low Risk group. Red is the positive correlation, blue is the negative correlation, and the depth of the color represents the strength of the correlation.

The process involved assessing immune infiltration in GC samples using the ssGSEA algorithm. It was carried out by computing the abundance of 28 immune cells in both risk groups based on the expression matrix acquired from TCGA-STAD. Firstly, the variation in the presence of immune cell infiltrates in various groups was illustrated by a group comparison plot. This diagram (**Figure 13A**) illustrated that the Activated CD4 T cells and Neutrophils of the two immune cells exhibited statistical significance (P < 0.05). Moreover, two immune cells, Eosinophils, and Activated dendritic cells, exhibited considerable statistical significance (P-value < 0.01). Furthermore, 20 immune cells, including Activated B cell, Activated CD8 T cell, Central memory CD8 T cell, Central memory CD4 T cell Effector memory CD8 T cell, Effector memory CD4 T cell etc, exhibited significant statistical significance (P < 0.001). The correlation heat map displayed the correlation outcomes of the abundance of 24 immune cell infiltrates in the two risk groups, as examined in the immune infiltration analysis (**Figures 13B, C**). The findings depicted that in the aforementioned groups of GC, most of the immune cells exhibited a positive relationship with each other. Finally, the correlation between the immune cell infiltration abundance and model genes was depicted through correlation bubble plots (**Figures 13D, E**). Within the cohort classified as high-risk, a significant positive correlation was identified between *NOX4* (the model gene) and Natural Killer T cells (immune cell) (P-value < 0.05, r value = 0.43). Conversely, *CD36* (model gene) depicted a negative correlation with Type 17 T helper cell (immune cell) (*P*-value < 0.05, r value = -0.29). In the low-risk group, *NOX4* (model gene) was noted to be positively correlated with Natural killer cells (immune cells) (P-value < 0.05, r value = 0.48). In contrast, *ANGPT2* (model gene) exhibited a negative association with Activated B cell (immune cell) (P-value < 0.05, r value = -0.24).

### 3.12 Immunogenicity score analysis

**Figure 14:**
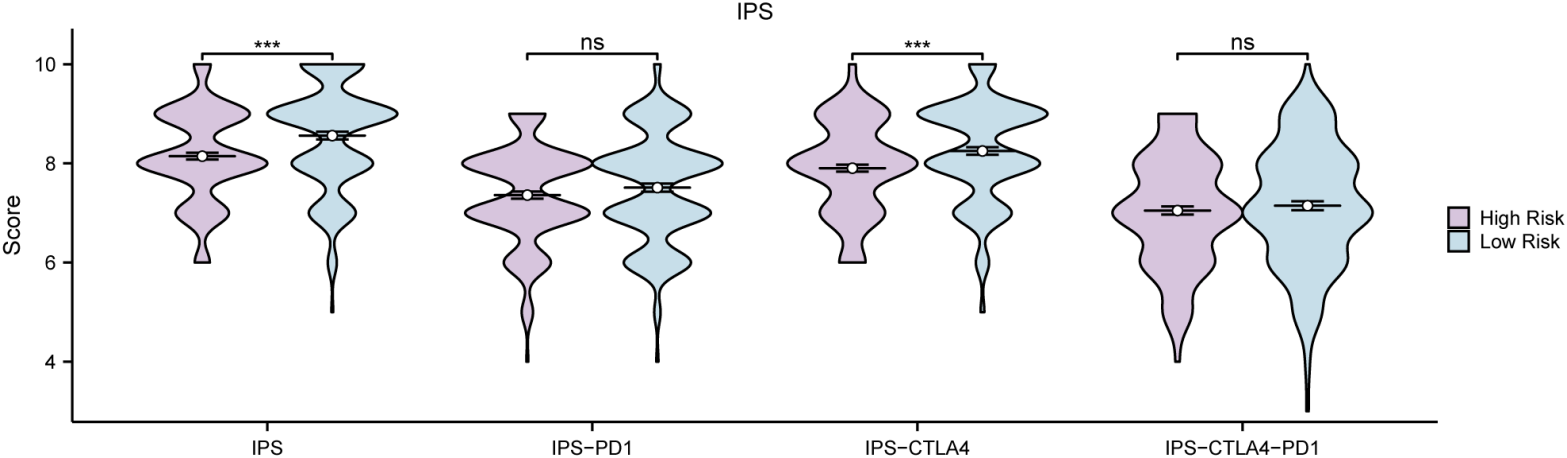
IPS Analysis Group comparison of immunogenicity scores (IPS) in the High Risk group and Low Risk group of gastric cancer (GC) samples from the gastric Cancer dataset (TCGA-STAD). TCGA, The Cancer Genome Atlas; STAD, Stomach Adenocarcinoma; GC, Gastric Cancer; IPS, Immunogenicity scores. ns stands for P value ≥ 0.01, not statistically significant; *** represents p value < 0.001, highly statistically significant. Purple represents the High Risk group and blue represents the Low Risk group.

Firstly, the GC samples obtained from TCGA-STAD were categorized into distinct groups based on the median LASSO Risk Score (RiskScore). Samples exhibiting values surpassing this median were designated as the high-risk group, whereas those with values falling below the median were classified as the low-risk group. To examine the prediction of immunotherapy in the aforementioned groups, IPS associated with GC samples were retrieved from the TCIA database. Furthermore, the R package ggplot2 was employed to generate the group comparison of various IPS in GC samples between the low-risk and high-risk groups of LASSO risk score (**Figure 14**). The results show that the IPS and IPS-CTLA4 classes exhibited high statistical significance between the two groups (P < 0.001).

### 3.13 The mRNA expression levels of genes involved in the prognostic signature

**Figure 15:**
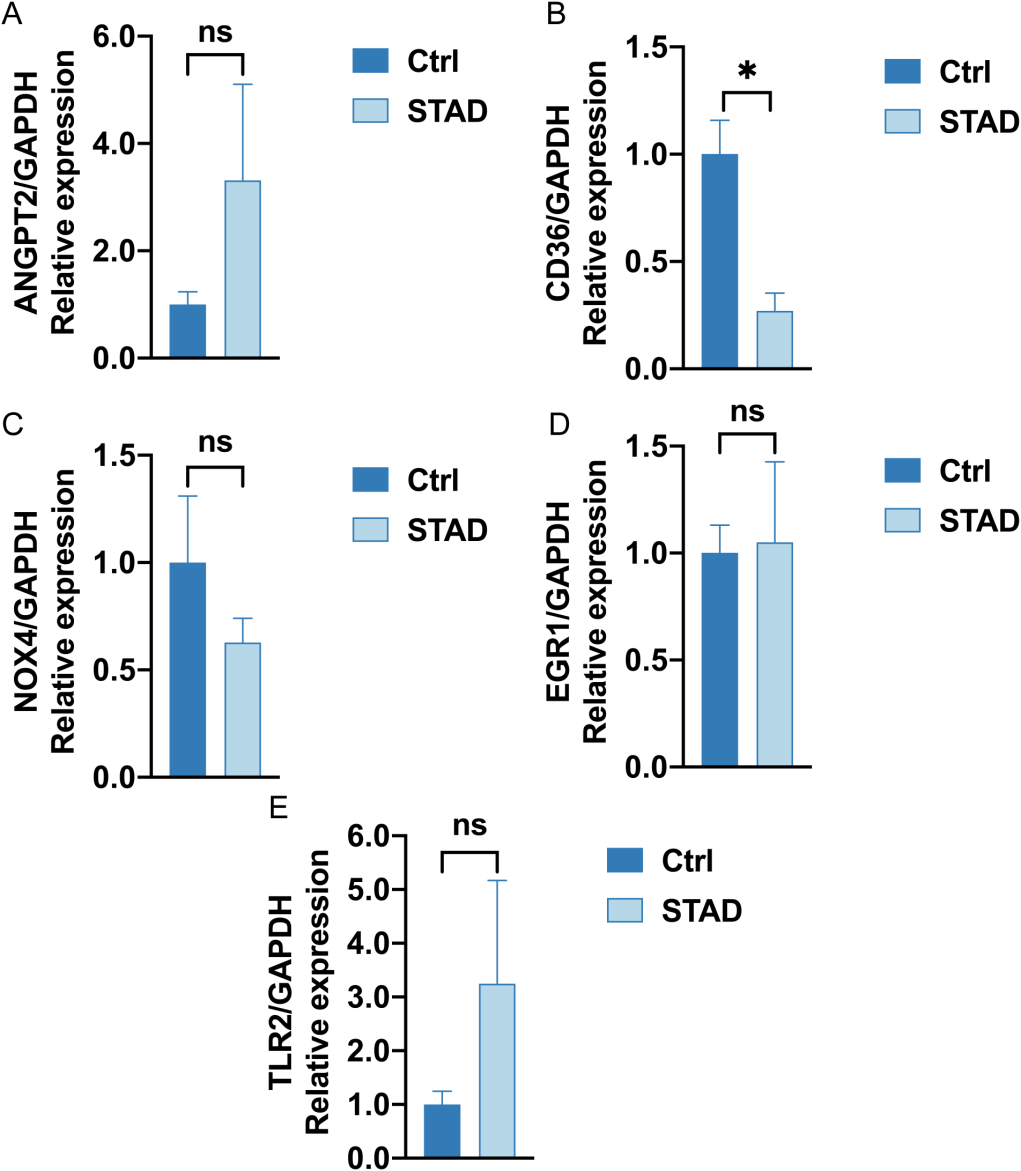
RT-qPCR analysis **(A–E)**. The mRNA expression of *ANGPT2, CD36, NOX4, EGR1, TLR2* in Ctrl group (left deep blue) and STAD group (right, baby blue). (ns stands for P value ≥ 0.05, not statistically significant; * represents P value < 0.05, statistically significant).

RT-qPCR results indicated that the mRNA expression levels of four hypoxic endoplasmic reticulum stress-related differential genes—*NOX4, ANGPT2, CD36*, and *TLR2*—were correlated with our established prognostic risk model in both gastric adenocarcinoma and adjacent normal tissues. The expression levels of *CD36* mRNA were notably decreased in gastric adenocarcinoma when compared to adjacent normal gastric tissues (P value < 0.05). *NOX4* expression showed a downward trend in gastric adenocarcinoma, although this was not statistically significant. In contrast, both *ANGPT2* and *TLR2* expressions exhibited upward trends, which also lacked statistical significance (**Figures 15A–E**). Increasing the sample size for these three genes may help achieve statistical significance for the observed differential results.

## 4 Discussion

GC remains among the most challenging malignancies, standing as the fifth most prevalent cancer and the fourth leading contributor to cancer-associated fatality globally (37). The gradual emergence and swift advancement of GC frequently result in diagnoses at an advanced stage, which considerably constrains available treatment alternatives and consequently diminishes survival rates (1). Despite advancements in adjuvant therapies and surgical techniques, the prognosis for GC patients remains poor, with a less than 30% 5-year survival rate observed in most countries (2). These findings emphasize the crucial requirement of therapeutic targets and novel biomarkers to enhance early detection, prognosis, and treatment strategies for GC.

The phenotypic complexity of GC, marked by its heterogeneity and multifactorial etiology, presents a significant challenge in the clinical setting (38). Recently, investigations have highlighted the influence of endoplasmic reticulum stress and hypoxia in tumorigenesis and tumor progression, suggesting that genes implicated in these pathways could offer promising potential biomarkers and treatment targets (4, 5, 39). This study first examines the interplay between hypoxia and ER stress-related genes in gastric cancer, aiming to elucidate their roles in disease prognosis. By integrating bioinformatics analyses with clinical data from TCGA and GEO, we identified 19 HERSRDEGs associated with hypoxia and ER stress that significantly impact overall survival in gastric cancer patients. Our findings indicate a potential for these genes to serve as biomarkers for early diagnosis and as targets for personalized therapeutic strategies, ultimately contributing to improved patient management.

The analysis of gene expression performed in our research identified a considerable quantity of differentially expressed genes (DEGs) linked to hypoxia and endoplasmic reticulum stress in GC. Specifically, the identification of 2762 DEGs, with 1279 upregulated and 1483 downregulated genes, underscores the complex molecular landscape of this malignancy. Among these, the selection of 19 HERSRDEGs such as *ANGPT2, ERG1, CD36, NOX4* and *TLR2*, provides critical insights into the potential roles these genes may play in gastric cancer pathophysiology. The findings highlight a need for further exploration into how these genes might serve as biomarkers for early detection and therapeutic targets. For instance, *ANGPT2*, also known as angiopoietin-2, is a protein that exerts a crucial influence on the modulation of angiogenesis and inflammation. Both of these processes are often dysregulated in cancer. Elevated levels of *ANGPT2* could lead to a more aggressive tumor phenotype and poorer prognosis by promoting vascular permeability and destabilization, thereby facilitating tumor cell dissemination (40, 41). *ANGPT2* plays a crucial role in the process of angiogenesis and possesses the ability to influence the immune response by altering the vascular environment in which immune cells reside (42). Studies have confirmed that in Hepatocellular Carcinoma (HCC), *AMYB* proto-oncogene-like 1(MYBL1) binds to the *ANGPT2* promoter and upregulates *ANGPT2* mRNA expression. This binding induces angiogenesis and makes HCC cells resistant to sorafenib; however, treatment with an *ANGPT2* monoclonal antibody significantly reduces the growth of tumors overexpressing *MYBL1* and effectively inhibits angiogenesis (43). The significant differential expression of *ANGPT2* observed in our research highlights its promise as both a therapeutic target and a prognostic biomarker, especially within the context of hypoxia and endoplasmic reticulum stress, which are known to exacerbate angiogenic signaling (44). Additionally, the relationship between these DEGs and other cancer types may reveal common pathways that could be targeted for broader therapeutic approaches. Inhibition of the integrin β-1 signaling pathway reliant on angiopoietin-2 significantly reduces the invasion and spread of small cell lung carcinoma (45). Blocking *ANGPT2* helps prevent pancreatic neuroendocrine tumors from spreading to the liver by increasing T-cell infiltration and stimulating the immune response (46). Monoclonal antibodies targeting *ANGPT2* have demonstrated efficacy in preclinical studies by blocking its action, leading to the inhibition of tumor angiogenesis and a reduction in tumor growth and metastasis.

*CD36* is a multifunctional glycoprotein involved in fatty acid metabolism, angiogenesis, and inflammation. It has been recognized as a facilitator of fatty acid uptake by cancer cells, thereby contributing to their energy supply and supporting rapid proliferation (47, 48). Moreover, *CD36* has been associated with metastatic processes in various cancers, including GC, where it might enhance the metastatic potential of cancer cells by promoting epithelial-mesenchymal transition (EMT) and tumor invasiveness (49). Research indicates that hypoxia within the peritoneal cavity promotes the expression of *CD36* in GC cells, thereby facilitating peritoneal metastasis via the absorption of free fatty acids. This study suggests that hypoxia-induced *CD36* expression may be one of the important mechanisms of gastric cancer progression (50). Monoclonal antibodies against *CD36* may reduce abdominal metastasis of gastric cancer, which is the focus of future preclinical research. Moreover, *CD36* has been associated with immune cell function, influencing the behavior of macrophage and dendritic cells (51). A recent study has shown that the *CD36-BATF2-MYB* axis may help predict the effectiveness of anti-PD-1 immunotherapy in treating gastric cancer (52). The univariate Cox regression analysis depicted the correlation between *CD36* and OS in GC patients. This correlation suggests that *CD36* might serve as a valuable prognostic indicator and could be explored as a target for therapeutic intervention, particularly in the modulation of tumor metabolism and immune response.

In this RT-qPCR validation, *CD36* demonstrated statistically significant variations in mRNA expression levels between gastric cancer tissues and adjacent healthy tissues. Furthermore, its expression level correlated with our established prognostic risk model, as shown in the **Figure 3A** volcano plot. Specifically, *CD36* mRNA expression was down-regulated in gastric cancer tissues compared to normal tissues. In the Cox prognostic analysis of TCGA-STAD, the hazard ratio (HR) for *CD36* was greater than 1, and the p-value was less than 0.05, indicating that higher expression of this gene is linked to a poorer prognosis. In our risk scoring model, the expression levels of CD36 were elevated in the high-risk cohort. Furthermore, the Kaplan-Meier survival analysis demonstrated that individuals exhibiting high CD36 expression experienced a less favorable prognosis. Several factors contribute to this pattern of expression and its differing biological functions. Genes have different roles in normal and disease states; in normal tissues, high expression of *CD36* helps to maintain normal physiological functions. In tumor tissues, on the other hand, high expression may promote tumor growth and spread, leading to a poor prognosis. Additionally, low *CD36* expression in tumors may result from gene mutations, epigenetic changes, or other regulatory factors. Therefore, further experimental studies are necessary to verify the specific mechanism.

The prognostic significance of these genes, particularly *ANGPT2, CD36, EGR1, NOX4*, and *TLR2*, which were highlighted in our univariate Cox regression analysis, suggests that they might serve as potential biomarkers for predicting patient outcomes. For instance, *CD36* has been associated with the metabolism of fatty acids and the spread of tumors, with its expression levels linked to unfavorable outcomes in various types of cancer (47). Similarly, the role of *NOX4* in generating ROS contributes to cellular signaling and survival, potentially affecting cancer progression (53). The current study utilized *NOX4* inhibitors to lower ROS levels, which may help slow down tumor progression (54). The development of a LASSO regression model incorporating *ANGPT2, CD36, and NOX4* further emphasizes the robustness of these genes as predictors of survival in GC patients. The model demonstrated that high-risk patients have substantially lower survival rates, suggesting that risk score could serve as a robust independent prognostic factor. Implementing this model in clinical settings could lead to timely interventions for high-risk patients, ultimately enhancing the management strategies for gastric cancer.

The potential challenges for clinical implementation of prognostic models are as follows: First, technical and logistical challenges. Accurately measuring the gene expression levels of the model requires high-quality biological samples. Therefore, it is critical to establish standardized operating procedures and quality control measures to ensure that tests are performed reliably across various healthcare facilities. Second, clinical workflow integration. Clinicians must learn how to apply the model in everyday diagnosis and treatment. This involves offering relevant application scenarios during patient examinations, follow-ups, and result interpretations. Third, there are potential obstacles to widespread clinical adoption of prognostic models. To promote the model, it is vital to address barriers by offering training, streamlining workflows, and showcasing the cost benefits of its adoption through economic assessments. Fourth, to improve the feasibility of implementing the model in clinics, conducting relevant pilot and real-world studies is essential. Thus, future research should prioritize validating the model in a large-scale, multicenter real-world setting to improve its reliability and practical applicability.

In this study, the analysis of functional enrichment pertaining to HERSRDEGs in GC demonstrated a substantial engagement in biological processes that are crucial for tumor advancement and cellular stress response. *CXCL8*, another HERDEGs, functions in the process of recruiting immune cells to the tumor microenvironment, potentially influencing anti-tumor immunity and tumor-promoting inflammation (55). The identification of *AURKA*, a gene involved in cell cycle regulation and mitotic spindle assembly, underscores the importance of cell proliferation in GC pathogenesis (56). GSEA results revealed several distinct signaling pathways and biological processes strongly linked to GC. These pathways include TP53 transcriptional regulation of cell cycle genes, photodynamic therapy-induced Nfkb survival signaling, TGF- β receptor signaling, and the influence of laminopathies On Wnt signaling. TP53, a recognized tumor suppressor gene, has an abnormal regulatory network linked to the development of numerous cancer types. Nfkb and TGF-β signaling pathways are pivotal in tumor progression. According to a study, peritoneal metastasis of GC was prevented by inhibiting EGR1/TGF-β1 (57). This finding contributes to the comprehension of the complex molecular mechanisms involved in the onset and progression of GC, potentially offering insights into novel therapeutic targets. Subsequent research should prioritize the validation of these pathways using experimental models, aiming to elucidate their functions in gastric cancer.

This study demonstrated notable disparities in the infiltration of immune cells, specifically regarding natural killer (NK) cells and T helper (Th) cells, when contrasting high-risk and low-risk cohorts of GC patients. NK cells, critical elements of the innate immune system, are acknowledged for their capability to detect and eliminate tumor cells without prior sensitization (58). Their cytotoxic activity is modulated by a balance of inhibitory and activating signals, susceptible to alteration within the tumor microenvironment (59). On the other hand, Th cells, particularly Th1 and Th17 subsets, have a critical involvement in orchestrating adaptive immune responses. Moreover, they have been involved in both tumor suppression and promotion, depending on the context (60). In this study, the immune infiltration and Immunogenicity score (IPS) analyses underscored the prognostic importance of the immune microenvironment in GC. High-risk patients exhibited a distinct immune profile in comparison with low-risk patients, suggesting that the failure of the immune system to recognize and eliminate cancer cells may contribute to a poorer prognosis (61). The differential immune landscapes between the risk groups could reflect the underlying mechanisms of immune escape and resistance to therapy, highlighting the potential for immunotherapeutic interventions. Research has demonstrated that in gastric cancer patients, the infiltration of regulatory T cells within the tumor microenvironment is associated with a poor prognosis, while the level of CD8+ T cell infiltration directly influences the patients’ treatment responses (62). Variations in immune cell infiltration within the tumor microenvironment can help evaluate cancer patients’ prognosis and forecast their response to immune checkpoint inhibitors.

Individuals with GC were categorized into two distinct groups (high- and low-risk) based on the constructed prognostic risk model. Subsequently, the relationship between various types of immune cell infiltrates and model genes was analyzed. *NOX4* showed a substantial positive association with Natural killer T cells (NKT) (r = 0.43) in the high-risk group. NKT cells are a distinct subset of immune cells that recognize lipid antigens and swiftly produce different cytokines, thus regulating the immune response. The activity of NKT cells may be inhibited in GC, which is significantly associated with the immune evasion strategies that operate within the tumor microenvironment. The acquired data implies that the increased *NOX4* expression is associated with the increased abundance of NKT cells in GC tissues of the high-risk group, possibly indicating some function of NKT cells in GC tissues or a specific immune microenvironment in GC. In the low-risk group, *NOX4* depicted a significantly positive relationship with Natural killer cells (NK) (r = 0.48). This suggests that the increased *NOX4* expression in low-risk GC tissues may be related to the presence of more NK cells. These NK cells exert a considerable influence on tumor defense, so it may be implied that high *NOX4* expression may reflect stronger immune surveillance. NK cell-based immunotherapy is a promising cancer treatment that utilizes the cytotoxic activity of NK cells against tumors. One approach involves using antibodies, like monalizumab, that block NKG2A and have shown potential to restore NK cell activity (63). A recent study on liver metastases in gastric cancer revealed that high levels of TGF-β in the tumor microenvironment lead to NK cell dysfunction. This dysfunction impairs the cytotoxicity of NK cells. The dysfunction of NK cells due to TGF-β may contribute to immune escape and the progression of gastric cancer. In the preclinical model, the therapeutic strategy of inhibiting TGF-β and enhancing NK cell activity demonstrated a strong antitumor effect. This combination therapy may effectively improve the prognosis of gastric cancer patients with liver metastases (64).This finding suggests that the immune contexture of the tumor microenvironment is modulated by the expression of HERSRDEGs, potentially impacting immunotherapy strategies and patient prognosis (65). The discovery of biomarkers associated with the immune response, along with the formulation of combination therapies aimed at both the tumor and its immune microenvironment, presents significant potential for the enhancement of GC treatment.

Despite the significant progress made in this study in constructing and evaluating a prognostic risk model for gastric cancer, there are still some limitations that need to be recognized. First. Geographical and ethnic differences. Geographic and ethnic differences in the gastric cancer samples from the TCGA and GEO databases may have influenced our findings. Such differences can result in variations in gene expression patterns, mutation frequencies, and their relationships with clinical outcomes, ultimately affecting the generalizability of our model. Future research directions should conduct prospective studies to validate the model in patient populations of different races, ages, genders, and pathological subtypes. Second. Clinical variables not included. Our model did not include clinical variables like patient lifestyle and treatment response, potentially limiting its predictive power. Future studies will investigate how these variables affect prognostic risk models, highlighting the need to include more clinical data to improve the accuracy and reliability of such models. Third. Limited sample size. Despite analyzing multiple datasets, our sample size was relatively small. This limitation may affect the statistical significance of our results. In the future, we plan to integrate data from different databases, especially samples from international collaborative projects, to enhance the broad applicability of our findings. Fourth. Insufficiency of functional mechanism discussion. Despite exhaustive cross-validation and multiple statistical analyses, the functional mechanisms of some biomarkers and pathways in our model have not been fully defined. Future experimental studies will aim to confirm the roles of key genes in gastric cancer progression using cell culture and gene manipulation techniques, while also exploring their molecular mechanisms. Future research will concentrate on the clinical application of our prognostic risk model, specifically evaluating its effectiveness in personalized treatment and prognostic assessments.

In summary, this study has identified GC-associated HERSRDEGs and has explored their roles in disease progression. Three HERSRDEGs model genes, including *ANGPT2*, *CD36*, and *NOX4*, showed significance in the development of a prognostic risk model for GC, suggesting their potential relevance to the survival outcomes of individuals with GC and their potential utility as prognostic biomarkers. This model effectively distinguishes between high- and low-risk GC groups. It shows a significant difference in immune cell infiltration between these patients, with high-risk patients having a lower IPS. These outcomes are crucial for elucidating GC molecular mechanisms and guiding personalized treatment. In their study of bladder cancer, Yaxuan Wang et al. (66–70) employed bioinformatics methods to uncover various biomarkers and mechanisms for diagnosis and prognosis, which assist in predicting disease outcomes and guiding treatment monitoring. These studies, together with our findings, indicate that bioinformatics can analyze genomic data to enhance the comprehension of disease mechanisms and has extensive applications in genome sequencing, mutation analysis, and personalized medicine. Bioinformatics algorithms can predict disease outcomes and optimize treatment strategies.

## Data Availability

The datasets presented in this study can be found in online repositories. The names of the repository/repositories and accession number(s) can be found in the article/**Supplementary Material**.
