# Supplementary material for: Prognostic significance and immune microenvironment infiltration patterns of hypoxia and endoplasmic reticulum stress-related genes in gastric cancer
Source: Front Oncol. 2025 Feb 21;15:1542740. doi: 10.3389/fonc.2025.1542740 (PMC11885130; doi:10.3389/fonc.2025.1542740)
Supplement: Supplementary file 1 [file DataSheet1.zip › Data Sheet 2/FIO-Supplementary-1/Negative results of bioinformatics analysis of gas.docx]

**Negative results of bioinformatics analysis of gastric cancer**

The RiskScore of gastric cancer (GC) samples in datasets GSE142000 and GSE118897 was calculated based on the risk coefficient of LASSO regression analysis. Gastric cancer (GC) samples of datasets GSE142000 and GSE118897 were divided into High Risk group and Low Risk group according to the median value of RiskScore expression. To explore the expression differences of Model Genes in gastric cancer (GC) samples from High Risk and Low Risk groups in datasets GSE142000 and GSE118897, The group comparison chart based on the expression of Model Genes (FigS1A-B) showed negative results.

**
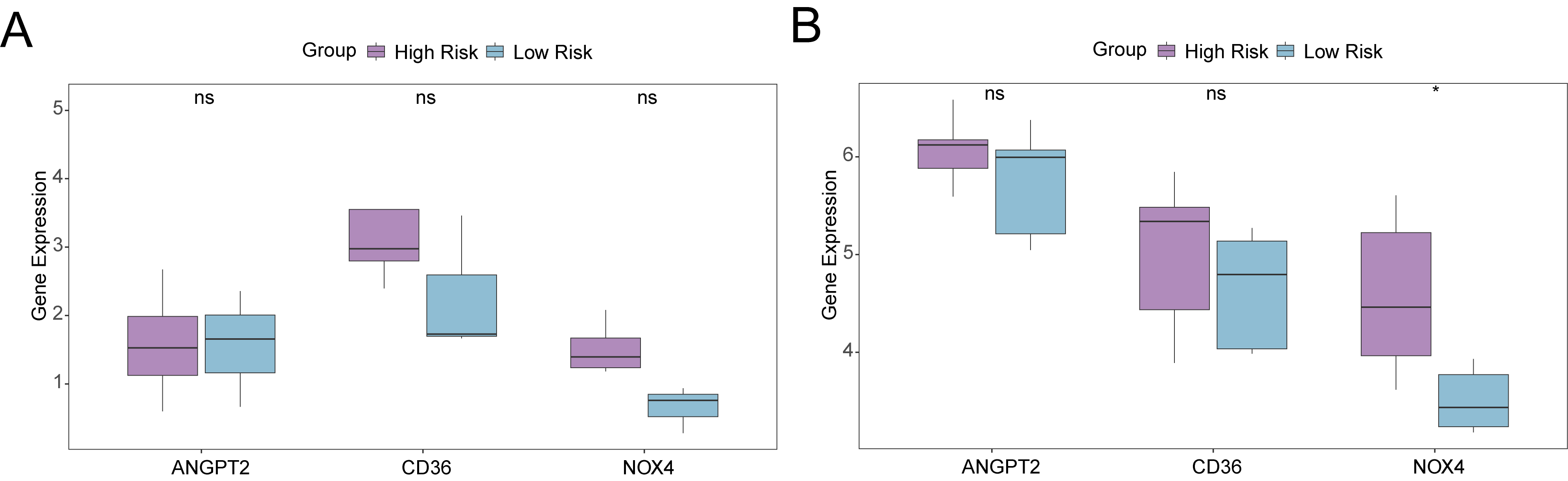
**

**Supplementary Fig.S1 Differential Expression Validation Analysis**

1. Group comparison diagram of Model Genes in High Risk group and Low Risk group of gastric cancer (GC) samples in dataset GSE142000. B. Group comparison diagram of Model Genes in High Risk group and Low Risk group of gastric cancer (GC) samples in dataset GSE118897. GC, Turner Syndrome. ns represents p value ≥ 0.05, no statistical significance; * represents a p value < 0.05, indicating statistical significance. The High Risk group is purple, and the Low Risk group is blue.
