# Supplementary material for: Prognostic significance and immune microenvironment infiltration patterns of hypoxia and endoplasmic reticulum stress-related genes in gastric cancer
Source: Front Oncol. 2025 Feb 21;15:1542740. doi: 10.3389/fonc.2025.1542740 (PMC11885130; doi:10.3389/fonc.2025.1542740)
Supplement: Supplementary file 1 [file DataSheet1.zip › Data Sheet 2/FIO-Supplementary-1/Supplementary TableS1 Baseline Table with GC Patients Characteristics.docx]

**Supplementary Table S1 Baseline Table with GC Patients Characteristics**

| Characteristics | overall |
| --- | --- |
| Age, n (%) |  |
| <= 60 | 113 (32.8%) |
| > 60 | 231 (67.2%) |
| Gender, n (%) |  |
| MALE | 218 (63.4%) |
| FEMALE | 126 (36.6%) |
| MStage, n (%) |  |
| M0 | 305 (88.7%) |
| M1 | 24 (7%) |
| MX | 15 (4.4%) |
| NStage, n (%) |  |
| N3 | 72 (20.9%) |
| NX | 5 (1.5%) |
| N0 | 107 (31.1%) |
| N1 | 88 (25.6%) |
| N2 | 72 (20.9%) |
| TStage, n (%) |  |
| T4 | 97 (28.2%) |
| T2 | 70 (20.3%) |
| T3 | 161 (46.8%) |
| T1 | 16 (4.7%) |
| Stage, n (%) |  |
| III | 148 (43%) |
| II | 110 (32%) |
| I | 49 (14.2%) |
| IV | 37 (10.8%) |

GC，Gastric Cancer。
