# Supplementary material for: Prognostic significance and immune microenvironment infiltration patterns of hypoxia and endoplasmic reticulum stress-related genes in gastric cancer
Source: Front Oncol. 2025 Feb 21;15:1542740. doi: 10.3389/fonc.2025.1542740 (PMC11885130; doi:10.3389/fonc.2025.1542740)
Supplement: Supplementary file 1 [file DataSheet1.zip › Data Sheet 2/FIO-Supplementary-1/Supplementary TableS10 qPCR primer sequence.docx]

**Supplementary TableS10 PCR Primer sequence**

| Primer name | **primer sequence(5'→3')** | PCR product**(bp)** |
| --- | --- | --- |
| H-GAPDH-F | GGAGCGAGATCCCTCCAAAAT | 197 |
| H-GAPDH-R | GGCTGTTGTCATACTTCTCATGG |  |
| H-NOX4-F | TGTGCCGAACACTCTTGGC | 136 |
| H-NOX4-R | ACATGCACGCCTGAGAAAATA |  |
| H-EGR1-F | GGTCAGTGGCCTAGTGAGC | 149 |
| H-EGR1-R | GTGCCGCTGAGTAAATGGGA |  |
| h-ANGPT2 (ANG2)-F | AACATCCCAGTCCACCTGAG | 202 |
| h-ANGPT2 (ANG2)-R | GGTCTTGCTTTGGTCCGTTA |  |
| H-CD36-F | CTTTGGCTTAATGAGACTGGGAC | 134 |
| H-CD36-R | GCAACAAACATCACCACACCA |  |
| h-TLR2-F | CTGCATTCCCAAGACACTGG | 131 |
| h-TLR2-R | GGGAGGCATCTGGTAGAGTC |  |
| H-NOX4-F | CAGATGTTGGGGCTAGGATTG | 96 |
| H-NOX4-R | GAGTGTTCGGCACATGGGTA |  |

**Note: The red primer is the first pair of primers, the yellow primer is the second pair of primers, and the final result is based on the result detected by the second pair of primers.**
