# Supplementary material for: Prognostic significance and immune microenvironment infiltration patterns of hypoxia and endoplasmic reticulum stress-related genes in gastric cancer
Source: Front Oncol. 2025 Feb 21;15:1542740. doi: 10.3389/fonc.2025.1542740 (PMC11885130; doi:10.3389/fonc.2025.1542740)
Supplement: Supplementary file 1 [file DataSheet1.zip › Data Sheet 2/FIO-Supplementary-1/Supplementary TableS2 GEO Datasets Information.docx]

**Supplementary Table S2 GEO Microarray Chip Information**

|  | GSE142000 | GSE118897 |
| --- | --- | --- |
| Platform | GPL23227 | GPL16686 |
| Species | Homo sapiens | Homo sapiens |
| Tissue | Gastric | Gastric |
| Samples in GC group | 7 | 10 |
| Samples in Control group | 6 | 10 |
| Reference | \ | PMID: 30404039 |

GEO，Gene Expression Omnibus；GC，Gastric Cancer。
