# Supplementary material for: Prognostic significance and immune microenvironment infiltration patterns of hypoxia and endoplasmic reticulum stress-related genes in gastric cancer
Source: Front Oncol. 2025 Feb 21;15:1542740. doi: 10.3389/fonc.2025.1542740 (PMC11885130; doi:10.3389/fonc.2025.1542740)
Supplement: Supplementary file 1 [file DataSheet1.zip › Data Sheet 2/FIO-Supplementary-1/Supplementary TableS3 GO KEGG Results.docx]

**Supplementary Table S3 Result of GO and KEGG Enrichment Analysis for HERSRDEGs**

| ONTOLOGY | ID | Description | GeneRatio | BgRatio | pvalue | p.adjust | qvalue |
| --- | --- | --- | --- | --- | --- | --- | --- |
| BP | GO:0050920 | regulation of chemotaxis | 6/19 | 225/18800 | 6.55E-08 | 7.50E-05 | 3.17E-05 |
| BP | GO:1902895 | positive regulation of miRNA transcription | 4/19 | 45/18800 | 1.08E-07 | 7.50E-05 | 3.17E-05 |
| BP | GO:0034599 | cellular response to oxidative stress | 6/19 | 284/18800 | 2.59E-07 | 7.50E-05 | 3.17E-05 |
| BP | GO:0034614 | cellular response to reactive oxygen species | 5/19 | 147/18800 | 2.91E-07 | 7.50E-05 | 3.17E-05 |
| BP | GO:0046686 | response to cadmium ion | 4/19 | 59/18800 | 3.27E-07 | 7.50E-05 | 3.17E-05 |
| CC | GO:0005788 | endoplasmic reticulum lumen | 4/19 | 311/19594 | 2.00E-04 | 8.56E-03 | 5.10E-03 |
| CC | GO:0045121 | membrane raft | 4/19 | 326/19594 | 2.39E-04 | 8.56E-03 | 5.10E-03 |
| CC | GO:0098857 | membrane microdomain | 4/19 | 327/19594 | 2.42E-04 | 8.56E-03 | 5.10E-03 |
| CC | GO:0030666 | endocytic vesicle membrane | 3/19 | 194/19594 | 8.24E-04 | 2.18E-02 | 1.30E-02 |
| CC | GO:0031526 | brush border membrane | 2/19 | 55/19594 | 1.28E-03 | 2.38E-02 | 1.42E-02 |
| MF | GO:0035325 | Toll-like receptor binding | 2/19 | 12/18410 | 6.62E-05 | 1.08E-02 | 5.43E-03 |
| MF | GO:0030169 | low-density lipoprotein particle binding | 2/19 | 18/18410 | 1.53E-04 | 1.25E-02 | 6.28E-03 |
| MF | GO:0070412 | R-SMAD binding | 2/19 | 24/18410 | 2.75E-04 | 1.25E-02 | 6.31E-03 |
| MF | GO:0038187 | pattern recognition receptor activity | 2/19 | 26/18410 | 3.23E-04 | 1.25E-02 | 6.31E-03 |
| MF | GO:0071813 | lipoprotein particle binding | 2/19 | 31/18410 | 4.61E-04 | 1.25E-02 | 6.31E-03 |
| KEGG | hsa04933 | AGE-RAGE signaling pathway in diabetic complications | 6/19 | 100/8164 | 6.93E-08 | 4.11E-06 | 1.92E-06 |
| KEGG | hsa05144 | Malaria | 5/19 | 50/8164 | 7.65E-08 | 4.11E-06 | 1.92E-06 |
| KEGG | hsa05142 | Chagas disease | 6/19 | 102/8164 | 7.80E-08 | 4.11E-06 | 1.92E-06 |
| KEGG | hsa05417 | Lipid and atherosclerosis | 7/19 | 215/8164 | 3.07E-07 | 1.21E-05 | 5.65E-06 |
| KEGG | hsa05171 | Coronavirus disease - COVID-19 | 7/19 | 232/8164 | 5.15E-07 | 1.63E-05 | 7.59E-06 |

GO，Gene Ontology；BP，Biological Process；CC，Cellular Component；MF，Molecular Function；KEGG，Kyoto Encyclopedia of Genes and Genomes；HERSRDEGs，Hypoxia&Endoplasmic Reticulum Stress-Related Differentially Expressed Genes。
