# Supplementary material for: Prognostic significance and immune microenvironment infiltration patterns of hypoxia and endoplasmic reticulum stress-related genes in gastric cancer
Source: Front Oncol. 2025 Feb 21;15:1542740. doi: 10.3389/fonc.2025.1542740 (PMC11885130; doi:10.3389/fonc.2025.1542740)
Supplement: Supplementary file 1 [file DataSheet1.zip › Data Sheet 2/FIO-Supplementary-1/Supplementary TableS4 GSEA Result.docx]

**Supplementary Table S4 Results of GSEA for TCGA-STAD**

| ID | Set Size | Enrichment Score | NES | p value |
| --- | --- | --- | --- | --- |
| REACTOME_CELL_CYCLE_CHECKPOINTS | 144 | 6.30E-01 | 2.45E+00 | 2.48E-03 |
| REACTOME_RESOLUTION_OF_SISTER_CHROMATID_COHESION | 71 | 6.92E-01 | 2.43E+00 | 2.31E-03 |
| REACTOME_CELL_CYCLE | 376 | 5.46E-01 | 2.37E+00 | 2.87E-03 |
| REACTOME_CELL_CYCLE_MITOTIC | 291 | 5.57E-01 | 2.36E+00 | 2.67E-03 |
| REACTOME_MITOTIC_PROMETAPHASE | 113 | 6.24E-01 | 2.35E+00 | 2.35E-03 |
| PID_PLK1_PATHWAY | 32 | 7.71E-01 | 2.28E+00 | 2.05E-03 |
| REACTOME_MITOTIC_SPINDLE_CHECKPOINT | 77 | 6.37E-01 | 2.25E+00 | 2.39E-03 |
| WP_RETINOBLASTOMA_GENE_IN_CANCER | 62 | 6.54E-01 | 2.23E+00 | 2.30E-03 |
| KEGG_CELL_CYCLE | 91 | 6.16E-01 | 2.23E+00 | 2.40E-03 |
| REACTOME_SEPARATION_OF_SISTER_CHROMATIDS | 92 | 6.12E-01 | 2.23E+00 | 2.37E-03 |
| REACTOME_MITOTIC_G1_PHASE_AND_G1_S_TRANSITION | 69 | 6.31E-01 | 2.21E+00 | 2.29E-03 |
| REACTOME_MITOTIC_METAPHASE_AND_ANAPHASE | 131 | 5.74E-01 | 2.20E+00 | 2.47E-03 |
| WP_CELL_CYCLE | 87 | 6.14E-01 | 2.20E+00 | 2.39E-03 |
| WP_DNA_IRDAMAGE_AND_CELLULAR_RESPONSE_VIA_ATR | 57 | 6.53E-01 | 2.19E+00 | 2.27E-03 |
| REACTOME_G2_M_CHECKPOINTS | 58 | 6.40E-01 | 2.15E+00 | 2.29E-03 |
| REACTOME_ACTIVATION_OF_ATR_IN_RESPONSE_TO_REPLICATION_STRESS | 24 | 7.71E-01 | 2.11E+00 | 2.11E-03 |
| REACTOME_TP53_REGULATES_TRANSCRIPTION_OF_CELL_CYCLE_GENES | 33 | 5.70E-01 | 1.70E+00 | 6.07E-03 |
| WP_PHOTODYNAMIC_THERAPYINDUCED_NFKB_SURVIVAL_SIGNALING | 24 | 5.99E-01 | 1.64E+00 | 8.44E-03 |
| WP_TGFBETA_RECEPTOR_SIGNALING | 41 | 5.15E-01 | 1.60E+00 | 8.64E-03 |
| WP_INFLUENCE_OF_LAMINOPATHIES_ON_WNT_SIGNALING | 18 | 5.99E-01 | 1.56E+00 | 2.49E-02 |

GSEA，Gene Set Enrichment Analysis；TCGA，The Cancer Genome Atlas；STAD，Stomach Adenocarcinoma。
