# Supplementary material for: Prognostic significance and immune microenvironment infiltration patterns of hypoxia and endoplasmic reticulum stress-related genes in gastric cancer
Source: Front Oncol. 2025 Feb 21;15:1542740. doi: 10.3389/fonc.2025.1542740 (PMC11885130; doi:10.3389/fonc.2025.1542740)
Supplement: Supplementary file 1 [file DataSheet1.zip › Data Sheet 2/FIO-Supplementary-1/Supplementary TableS5 Cox Result.docx]

**Supplementary Table S5 Results of Cox Analysis**

| Characteristics | Total(N) | Univariate analysis | | Multivariate analysis | |
| --- | --- | --- | --- | --- | --- |
|  |  | HR (95% CI) | P value | HR(95% CI) | P value |
| Age | 344 |  |  |  |  |
| <= 60 | 113 | Reference |  | Reference |  |
| > 60 | 231 | 1.646 (1.119 - 2.422) | 0.011 | 1.800 (1.208 - 2.682) | 0.004 |
| Gender | 344 |  |  |  |  |
| MALE | 218 | Reference |  |  |  |
| FEMALE | 126 | 0.802 (0.558 - 1.153) | 0.233 |  |  |
| MStage | 344 |  |  |  |  |
| M0 | 305 | Reference |  | Reference |  |
| M1 | 24 | 2.088 (1.175 - 3.711) | 0.012 | 1.032 (0.437 - 2.439) | 0.943 |
| MX | 15 | 1.256 (0.512 - 3.077) | 0.618 | 1.315 (0.514 - 3.367) | 0.568 |
| NStage | 344 |  |  |  |  |
| N3 | 72 | Reference |  | Reference |  |
| NX | 5 | 0.395 (0.054 - 2.874) | 0.359 | 0.656 (0.081 - 5.331) | 0.693 |
| N1 | 88 | 0.574 (0.367 - 0.898) | 0.015 | 0.730 (0.429 - 1.243) | 0.246 |
| N0 | 107 | 0.381 (0.236 - 0.618) | < 0.001 | 0.592 (0.255 - 1.375) | 0.223 |
| N2 | 72 | 0.604 (0.373 - 0.979) | 0.041 | 0.807 (0.484 - 1.348) | 0.413 |
| TStage | 344 |  |  |  |  |
| T4 | 97 | Reference |  | Reference |  |
| T3 | 161 | 0.971 (0.656 - 1.438) | 0.884 | 1.056 (0.690 - 1.614) | 0.803 |
| T2 | 70 | 0.739 (0.445 - 1.228) | 0.243 | 0.982 (0.475 - 2.031) | 0.961 |
| T1 | 16 | 0.116 (0.016 - 0.847) | 0.034 | 0.248 (0.028 - 2.209) | 0.211 |
| Stage | 344 |  |  |  |  |
| III | 148 | Reference |  | Reference |  |
| II | 110 | 0.690 (0.456 - 1.043) | 0.078 | 0.883 (0.449 - 1.735) | 0.718 |
| IV | 37 | 1.807 (1.123 - 2.910) | 0.015 | 2.085 (1.028 - 4.227) | 0.042 |
| I | 49 | 0.431 (0.227 - 0.817) | 0.010 | 0.841 (0.223 - 3.171) | 0.799 |
| Risk.Score | 344 | 4.861 (2.199 - 10.744) | < 0.001 | 3.891 (1.717 - 8.817) | 0.001 |

The hazard ratio (HR) is a statistical measure. An HR greater than 1 indicates a risk factor, while an HR less than 1 indicates a protective factor.We included factors with a p-value of less than 0.1 in the analysis.
