# Supplementary material for: Prognostic significance and immune microenvironment infiltration patterns of hypoxia and endoplasmic reticulum stress-related genes in gastric cancer
Source: Front Oncol. 2025 Feb 21;15:1542740. doi: 10.3389/fonc.2025.1542740 (PMC11885130; doi:10.3389/fonc.2025.1542740)
Supplement: Supplementary file 1 [file DataSheet1.zip › Data Sheet 2/FIO-Supplementary-1/Supplementary TableS6 GSVA for Risk Group.docx]

**Supplementary Table S6 Results of GSVA for Risk Group**

| ID | logFC | AveExpr | p value | adj.p value |
| --- | --- | --- | --- | --- |
| HALLMARK_MYC_TARGETS_V2 | 2.86E-01 | -1.09E-02 | 6.35E-11 | 1.98E-10 |
| HALLMARK_E2F_TARGETS | 2.72E-01 | 2.16E-03 | 3.92E-10 | 1.15E-09 |
| HALLMARK_G2M_CHECKPOINT | 2.66E-01 | 4.46E-03 | 1.61E-11 | 5.75E-11 |
| HALLMARK_MYC_TARGETS_V1 | 2.56E-01 | -1.51E-02 | 1.92E-09 | 4.00E-09 |
| HALLMARK_OXIDATIVE_PHOSPHORYLATION | 2.15E-01 | -2.13E-02 | 2.29E-08 | 4.59E-08 |
| HALLMARK_DNA_REPAIR | 2.14E-01 | -2.25E-02 | 4.37E-11 | 1.46E-10 |
| HALLMARK_MTORC1_SIGNALING | 2.08E-01 | -2.35E-03 | 1.42E-09 | 3.22E-09 |
| HALLMARK_PEROXISOME | 1.85E-01 | 8.09E-04 | 9.54E-10 | 2.51E-09 |
| HALLMARK_UNFOLDED_PROTEIN_RESPONSE | 1.71E-01 | -7.88E-03 | 4.34E-08 | 8.35E-08 |
| HALLMARK_FATTY_ACID_METABOLISM | 1.48E-01 | -1.23E-02 | 8.94E-07 | 1.54E-06 |
| HALLMARK_IL6_JAK_STAT3_SIGNALING | -2.14E-01 | -8.98E-04 | 7.59E-10 | 2.11E-09 |
| HALLMARK_ALLOGRAFT_REJECTION | -2.17E-01 | 3.87E-03 | 1.71E-09 | 3.71E-09 |
| HALLMARK_APICAL_JUNCTION | -2.34E-01 | -1.00E-02 | 2.11E-19 | 2.11E-18 |
| HALLMARK_MYOGENESIS | -2.40E-01 | -1.39E-02 | 8.53E-16 | 5.33E-15 |
| HALLMARK_KRAS_SIGNALING_UP | -2.69E-01 | -7.35E-03 | 1.26E-25 | 3.16E-24 |
| HALLMARK_HEDGEHOG_SIGNALING | -2.73E-01 | -7.68E-03 | 1.12E-16 | 8.00E-16 |
| HALLMARK_INFLAMMATORY_RESPONSE | -2.77E-01 | -1.72E-03 | 7.65E-17 | 6.37E-16 |
| HALLMARK_UV_RESPONSE_DN | -2.83E-01 | -2.09E-02 | 9.43E-24 | 1.18E-22 |
| HALLMARK_ANGIOGENESIS | -3.44E-01 | -7.90E-03 | 5.93E-24 | 9.88E-23 |
| HALLMARK_EPITHELIAL_MESENCHYMAL_TRANSITION | -5.16E-01 | -1.28E-02 | 1.96E-44 | 9.82E-43 |

GSVA，Gene Set Variation Analysis。
