# Supplementary figures and images for: Prognostic significance and immune microenvironment infiltration patterns of hypoxia and endoplasmic reticulum stress-related genes in gastric cancer
Source: Front Oncol. 2025 Feb 21;15:1542740. doi: 10.3389/fonc.2025.1542740 (PMC11885130; doi:10.3389/fonc.2025.1542740)

A

Group 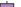 High Risk 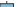 Low Risk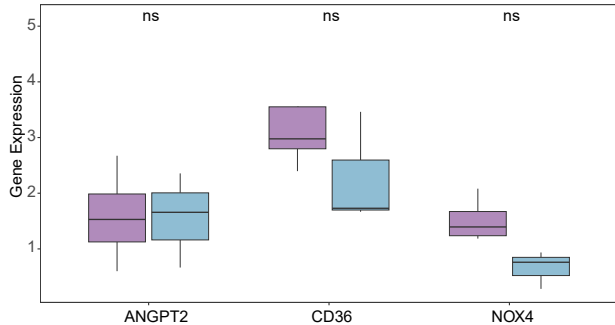

B

Group 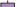 High Risk 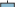 Low Risk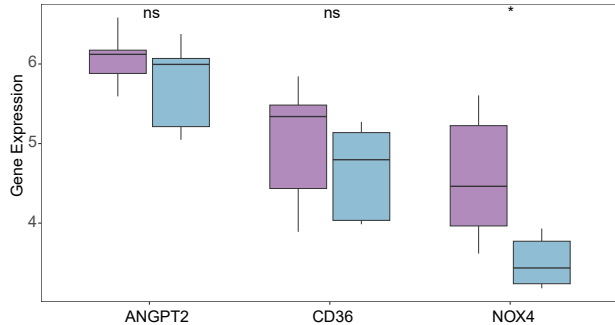

Supplement: Supplementary file 1 [file DataSheet1.zip › Data Sheet 2/FIO-Supplementary-1/Supplementary Figure S1. Comparison of Model Genes Between High-Risk and Low-Risk Groups in Gastric Cancer (GC) Samples Across Dataset GSE142000(Fig 1A) and GSE118897(Fig 1B).pdf]
